# Changing epidemiological patterns of tuberculosis in China from 1990 to 2023: trends and future projections of drug-resistant and HIV-associated tuberculosis

**DOI:** 10.3389/fpubh.2026.1948171

**Published:** 2026-09-18

**Authors:** Jiajun Chen, Peiyong Zheng, Qinyan Zuo, Shaoyan Zhang, Lei Qiu, Xianwei Wu, Zhenhui Lu

**Affiliations:** 1 Institute of Respiratory Diseases, Longhua Hospital, Shanghai University of Traditional Chinese Medicine, Shanghai, China; 2 Clinical Medicine Center, Longhua Hospital, Shanghai University of Traditional Chinese Medicine, Shanghai, China

**Keywords:** China, exponential smoothing, extensively drug-resistant TB, HIV-associated TB, projection, tuberculosis

## Abstract

**Background:**

China has made substantial progress in tuberculosis (TB) control over the past decades. However, continued surveillance of drug-resistant and HIV-associated TB remains essential for optimizing future TB control strategies. This study aimed to comprehensively assess the long-term burden and future trends of TB and its major subtypes in China, with particular emphasis on extensively drug-resistant TB (XDR-TB) and HIV-associated TB.

**Methods:**

Data on incidence, prevalence, mortality, and disability-adjusted life years (DALYs) of TB in China from 1990 to 2023 were extracted from the Global Burden of Disease Study 2023. Historical age-standardized rates were reported with 95% uncertainty intervals, and temporal trends in HIV-negative TB, drug-susceptible TB (DS-TB), multidrug-resistant TB (MDR-TB), XDR-TB, and HIV-associated TB subtypes were quantified using average annual percent changes (AAPCs) with 95% confidence intervals. Log-transformed age-standardized rates were projected from 2024 to 2035 using damped-trend exponential smoothing, ETS(A, Ad, N), with the damping parameter selected by rolling-origin validation. Forecasts were reported with 95% prediction intervals; projections to 2050 were treated as exploratory and reported in the Supplementary material.

**Results:**

From 1990 to 2023, the age-standardized incidence rates of HIV-negative TB, DS-TB, and MDR-TB declined overall in China, with AAPCs of −3.833, −3.877, and −2.859%, respectively. In contrast, XDR-TB increased markedly (AAPC 7.134%), while all HIV-associated TB subtypes also showed upward trends, particularly HIV-XDR-TB, which exhibited the fastest increase in incidence (AAPC 13.318%). Recent trends were more heterogeneous, including increases in HIV-negative TB and DS-TB incidence during 2019–2023 and declining or stable mortality trends for several drug-resistant and HIV-associated subtypes. By 2035, the incidence and prevalence of XDR-TB, HIV-DS-TB, and HIV-MDR-TB were projected to increase modestly, whereas their mortality and DALY rates were projected to decline. The XDR-TB incidence rate was projected to reach 0.172 per 100,000 population (95% prediction interval: 0.077–0.381). HIV-XDR-TB incidence was projected to remain broadly stable, with a slight decline, whereas its prevalence was projected to increase. Prediction intervals were wide for several low-burden drug-resistant outcomes.

**Conclusion:**

Although the overall TB burden in China has declined substantially, subtype-specific trajectories remain heterogeneous. Future control strategies should strengthen the rapid detection and management of drug resistance, integrate TB and HIV services, and prioritize targeted prevention and care for high-risk populations.

## Background

Tuberculosis (TB) is a chronic and potentially debilitating infectious disease caused by *Mycobacterium tuberculosis* (MTB), most commonly affecting the lungs but capable of involving almost any organ (1, 2). TB is primarily transmitted through airborne particles released by individuals with infectious pulmonary TB. Globally, MTB infection is widespread, and most infected individuals remain asymptomatic and non-infectious. However, latent infection may progress to active TB when host immune defenses are weakened. The typical clinical manifestations of active TB include a prolonged cough, sputum production, hemoptysis, fever, night sweats, weight loss, and fatigue. Extrapulmonary TB presents with symptoms specific to the affected organs (3–6). If left untreated, TB can lead to pulmonary fibrosis, respiratory failure, and even death, significantly increasing the morbidity and mortality risks, especially among immunocompromised populations, such as people living with HIV (6–8). Furthermore, the spread of drug-resistant TB (e.g., MDR-TB and XDR-TB) complicates treatment and exacerbates both the individual and societal economic burden (2, 9, 10).

According to the World Health Organization’s Global Tuberculosis Report 2024, TB remains the leading infectious cause of death worldwide. In 2023, there were approximately 10.8 million new cases and 1.25 million deaths globally, with the disease burden concentrated in low- and middle-income countries in Southeast Asia, Africa, and the Western Pacific (1, 5, 11–13). Although TB incidence and mortality rates have shown a gradual decline, progress in control has not yet reached the World Health Organization’s 2025 milestones. This indicates that global TB control efforts continue to face multiple challenges, including insufficient funding, severe drug resistance, and the economic burden on patients (1, 5, 11–13).

In China, TB control has achieved some success through a “three-in-one” service system, but substantial challenges remain. In 2023, approximately 741,000 new TB cases were reported (3). Despite efforts, several challenges persist in China’s TB control system: case detection still relies substantially on passive presentation to healthcare facilities, resulting in high transmission risks before diagnosis; there is a large pool of people with latent infections, making preventive treatment difficult; uneven capacity for TB prevention and control across regions, coupled with inadequate policy and legal support in certain areas; and insufficient funding mechanisms, with preventive treatment not yet covered by health insurance, further burdening patients economically. These systemic issues significantly hinder China’s ability to achieve the goals outlined in the “End TB Strategy” (5, 11, 14).

Previous GBD-based studies have primarily focused on the overall burden of tuberculosis or were based on earlier GBD releases. Comprehensive evaluations of long-term trends in drug-resistant tuberculosis, particularly XDR-TB, together with HIV-associated tuberculosis and future projections using the updated GBD 2023 database remain limited. Therefore, an updated assessment is warranted to better inform TB control priorities in China. This study, through a comprehensive analysis of the GBD 2023 data, systematically evaluates TB prevalence and trends in China. It explores the trends in disease burden, the epidemiology of drug-resistant TB, and the status of HIV-associated TB. The findings will help identify key weaknesses in TB control efforts and support the implementation of more effective, targeted interventions, ultimately aiming to reduce TB transmission and mortality and achieve the goal of “Ending TB.”

## Methods

### Data sources

The data for this study were sourced from the GBD 2023 database,^1^ curated by the Institute for Health Metrics and Evaluation (IHME) at the University of Washington. The GBD 2023 study provides assessments for 204 countries and territories, covering 375 diseases and 88 risk factors (15–17). We extracted aggregated estimates for China from 1990 to 2023 by year, sex, measure, and metric. The measures included incidence, prevalence, deaths, and disability-adjusted life years (DALYs), reported as absolute numbers and rates. The causes included overall tuberculosis, drug-susceptible tuberculosis (DS-TB), multidrug-resistant tuberculosis without extensive drug resistance (MDR-TB), extensively drug-resistant tuberculosis (XDR-TB), and the corresponding HIV-associated TB subtypes.

For China, TB-specific metrics (1990–2023) included incidence, prevalence, mortality, and DALYs, reported as age-standardized rates (ASR) with 95% uncertainty intervals (UI), including age standardized incidence rate (ASIR), age standardized prevalence rate (ASPR), age standardized mortality rate (ASMR) and age standardized DALY rate (ASDR), alongside absolute counts (incidences, prevalences, deaths, DALYs). Estimates were generated using DisMod-MR 2.1 and meta-regression with Bayesian priors, regularization, and trimming (MR-BRT) models. In GBD 2023, cause-of-death data for China primarily come from the China Cause of Death Monitoring System, the China Maternal and Child Health Monitoring Network, the China Centers for Disease Control and Prevention (CDC) cause-of-death reports, and census data. Incidence and prevalence data are derived from national disease surveillance sites, the National Health Services Survey, and relevant published literature. Disability-adjusted life years (DALY) data for diseases resulting from disability rely on hospital discharge databases, household health surveys, and cohort study data (12, 13, 15). These national data systems served as upstream inputs to the GBD estimation process; the present study analyzed the aggregated GBD 2023 estimates and did not directly reanalyze individual-level records from these systems. Detailed GBD 2023 data extraction parameters, cause and subcause identifiers, and the tuberculosis classification hierarchy are provided in Supplementary methods S2.

### Definitions

In accordance with the GBD 2023 cause hierarchy and recent GBD-based studies of tuberculosis burden, TB was classified according to HIV status and drug-resistance category (18–21). HIV-negative active TB was further categorized as drug-susceptible tuberculosis (DS-TB), multidrug-resistant tuberculosis without extensive drug resistance (MDR-TB), and extensively drug-resistant tuberculosis (XDR-TB). Correspondingly, HIV-associated active TB was categorized as HIV-associated drug-susceptible tuberculosis (HIV-DS-TB), HIV-associated multidrug-resistant tuberculosis without extensive drug resistance (HIV-MDR-TB), and HIV-associated extensively drug-resistant tuberculosis (HIV-XDR-TB). Under the GBD 2023 case definitions, DS-TB was defined as TB susceptible to isoniazid and rifampicin; MDR-TB referred specifically to TB resistant to isoniazid and rifampicin but not meeting the GBD 2023 definition of XDR-TB; and XDR-TB was defined as TB resistant to isoniazid and rifampicin, plus any fluoroquinolone and at least 1 s-line injectable drug (amikacin, kanamycin, or capreomycin). The corresponding drug-resistance definitions were applied to HIV-associated TB categories. Within each HIV stratum, these active-TB drug-resistance categories were mutually exclusive by construction and together represented the corresponding modeled active TB categories in the GBD 2023 hierarchy. These cause categories were retained as defined in GBD 2023 to ensure consistency across years and burden measures.

For the identification of TB in the GBD 2023 framework, the relevant ICD-10: A10-A19.9, B90-B90.9, K67.3, K93.0, M49.0, P37.0 and the corresponding ICD-9 codes were 010–019.9, 137–137.9, 138.0, 138.9, 139.9, 320.4, 730.4–730.6. HIV-TB co-infection was identified using the ICD-10 code B20.0 (15). Detailed GBD 2023 case definitions are provided in Supplementary methods S2.

### Statistical analysis

The disease burden of tuberculosis is assessed using both relative and absolute indicators. Relative burden was expressed through age-standardized rates, including incidence, prevalence, mortality, and disability-adjusted life years (DALYs) rates, all per 100,000 population. Absolute burden was measured by actual case numbers, covering incident cases, prevalent cases, deaths, and DALYs. Historical GBD estimates were reported with 95% uncertainty intervals (UIs). Annual percentage changes (APCs) and average annual percent changes (AAPCs) were reported with 95% confidence intervals (CIs), whereas ETS forecast estimates were reported with 95% prediction intervals (PIs). Historical trends were assessed using Joinpoint regression, and annual age-standardized rates were projected using log-scale damped-trend exponential smoothing, ETS(A, Ad, N) (22–26). Forecast-period AAPCs were calculated from the projected point estimates and reported with conditional 95% CIs, which did not incorporate the full uncertainty of the forecasts.

### Joinpoint regression analysis

The annual percent change (APC), average annual percent change (AAPC) and its corresponding 95% confidence interval (CI) were used to quantify temporal trends (27–29). The formula for calculating APC and AAPC is as follows:


$$\mathrm{AP}C_{i}=\{\exp(\mathrm{\beta i})-1\}\times 100$$

$$\mathrm{AAP}C_{i}=\{\exp(\frac{\sum W_{i}\beta_{i}}{\sum W_{i}})-1\}\times 100$$

Historical trends were fitted using log-linear models, with calendar year as the independent variable and the natural logarithm of the ASR as the dependent variable. To account for heteroscedasticity, the annual standard error of each ASR was approximated as (upper 95% UI − lower 95% UI)/(2 × 1.96). The number and location of joinpoints were identified using the Grid Search Method. Models with 0–5 joinpoints were evaluated, with at least two observations required between adjacent joinpoints and between each joinpoint and either endpoint of the series. The optimal model was selected using the weighted Bayesian information criterion (WBIC), with lower values indicating better fit. In the model, i represents the number of segments, while $\beta_{i}$ denotes the regression coefficients for each linear segment. The length of each segment is represented by $W_{i}$ . If the lower bound of the 95% CI for the AAPC is greater than 0 and *p* < 0.05, this indicates an increasing trend. Conversely, if the upper bound of the 95% CI is less than 0 and *p* < 0.05, this indicates a decreasing trend (27–29). Historical trend analyses were performed using R version 4.4.2.

### ETS forecasting analysis

Annual age-standardized rates (ASRs) for each TB subtype and burden measure from 1990 to 2023 were projected using a damped-trend exponential smoothing model corresponding to an ETS(A, Ad, N) specification with additive errors, a damped additive trend, and no seasonal component (23, 24). Each series was modeled on the natural logarithmic scale to stabilize variance and ensure positive back-transformed forecasts. The damping parameter was selected separately for each series from candidate values of 0.80, 0.85, 0.90, 0.93, and 0.95 using rolling-origin validation across six training endpoints (2013–2018), each evaluated over the subsequent 5 years; the value with the lowest pooled root mean squared error on the logarithmic scale was retained (25). The 95% prediction intervals (PIs) were calculated on the logarithmic scale and subsequently back-transformed (26).

Projections were generated for 2024–2050 using a single fitted trajectory. The year 2035 was designated as the primary endpoint because it corresponds to the target year of the WHO End TB Strategy and involves less long-range extrapolation, whereas projections to 2050 were treated as exploratory and are reported in the Supplementary material. Forecast-period average annual percent changes (AAPCs) were estimated by segmented log-linear regression of the projected point estimates, allowing up to one joinpoint for 2024–2035 and three for 2024–2050, with at least 5 years between segment boundaries and model selection based on the Bayesian information criterion. Sensitivity analyses using alternative historical observation windows were conducted for XDR-TB DALYs because of the greater variability of this series. Forecast generation and forecast-period trend analyses were implemented using custom Python scripts (Python 3.12.13; NumPy 2.3.5; pandas 2.2.3; SciPy 1.17.0). Detailed ETS model specification, validation procedures, prediction interval estimation, and forecast-period trend analyses are provided in Supplementary methods S1.

## Results

### HIV-negative TB and drug-susceptible TB

In 2023, the ASIR of HIV-negative TB in China was 38.430 per 100,000 population (95% UI: 34.008–42.938), corresponding to 652,167 incident cases (95% UI: 581,880–722,670). The ASMR and ASDR were 1.170 (95% UI: 0.864–1.640) and 54.233 (95% UI: 42.908–70.143) per 100,000, respectively, corresponding to 24,752 deaths and 990,704 DALYs. The prevalence estimate for TB, which included latent tuberculosis infection, was 30,714.913 per 100,000 population (95% UI: 27,838.234–33,598.704), corresponding to approximately 494.2 million prevalent cases. DS-TB accounted for most of the active TB burden, with an ASIR of 36.656, an ASPR of 63.377, an ASMR of 1.054, and an ASDR of 49.904 per 100,000 population in 2023 (Table 1 and Supplementary Tables S1–S6).

**Table 1:** Trends in the disease burden of tuberculosis and its subtypes in China from 1990 to 2023.

Diseases	Incidence		Prevalence		Death		DALYs	
	ASIR (per 100, 000 populations, 95% UI). 2023.	AAPC (95% CI). 1990–2023.	ASPR (per 100, 000 populations, 95% UI). 2023.	AAPC (95% CI). 1990–2023.	ASMR (per 100, 000 populations, 95% UI). 2023.	AAPC (95% CI). 1990–2023.	ASDR (per 100,000 populations, 95%UI). 2023.	AAPC (95% CI). 1990–2023.
TB	38.430(34.008, 42.938)	−3.833 (−3.995, −3.671)	30714.913(27838.234, 33598.704)	−0.094 (−0.133, −0.054)	1.170(0.864, 1.640)	−8.183 (−8.482, −7.885)	54.233(42.908, 70.143)	−7.501 (−7.782, −7.221)
Male	47.997(43.003, 53.058)	−3.490 (−3.653, −3.328)	31765.802(28815.571, 34536.162)	−0.168 (−0.235, −0.101)	1.789(1.306, 2.559)	−7.747 (−8.094, −7.399)	77.038(60.984, 98.433)	−7.145 (−7.369, −6.922)
Female	29.015(25.323, 32.927)	−4.307 (−4.463, −4.152)	29625.704(26764.269, 32581.274)	−0.214 (−0.285, −0.143)	0.605(0.386, 1.236)	−9.249 (−9.563, −8.935)	31.732(23.210, 52.107)	−8.191 (−8.429, −7.953)
HIV	2.360(1.244, 3.994)	3.787 (3.317, 4.257)	31.444(16.508, 59.429)	6.189 (6.037, 6.342)	1.648(1.148, 2.171)	7.900 (7.018, 8.783)	78.206(54.701, 104.913)	7.776 (7.025, 8.528)
Male	3.539(1.894, 5.900)	4.165 (3.375, 4.955)	46.843(24.246, 88.580)	6.353 (6.189, 6.517)	2.510(1.751, 3.322)	8.387 (7.621, 9.153)	116.015(81.095, 156.405)	8.213 (7.586, 8.841)
Female	1.077(0.572, 1.876)	2.652 (2.126, 3.178)	15.553(8.294, 30.095)	5.614 (5.468, 5.760)	0.769(0.533, 1.056)	6.805 (5.903, 7.706)	38.469(25.905, 54.754)	6.625 (5.875, 7.374)
DS-TB	36.656(30.724, 41.688)	−3.877 (−3.967, −3.788)	63.377(54.494, 71.783)	−3.369 (−3.608, −3.130)	1.054(0.715, 1.551)	−8.332 (−8.602, −8.062)	49.904(36.097, 66.190)	−7.460 (−7.680, −7.240)
Male	45.768(38.639, 51.707)	−3.500 (−3.674, −3.326)	84.841(72.506, 95.961)	−3.148 (−3.479, −2.818)	1.611(1.078, 2.372)	−7.683 (−7.966, −7.400)	70.789(52.527, 93.608)	−7.094 (−7.340, −6.847)
Female	27.691(23.049, 31.807)	−4.338 (−4.441, −4.236)	42.153(36.017, 48.503)	−3.920 (−4.088, −3.753)	0.545(0.310, 1.112)	−9.241 (−9.487, −8.996),	29.294(20.012, 48.345)	−8.146 (−8.330, −7.962)
MDR-TB	1.630(0.196, 4.967)	−2.859 (−3.487, −2.231)	2.740(0.340, 8.794)	−1.628 (−2.410, −0.846)	0.098(0.011, 0.354)	−6.714 (−7.265, −6.164)	3.718(0.466, 13.091)	−6.633 (−7.130, −6.135)
Male	2.049(0.248, 6.275)	−2.361 (−3.065, −1.657)	3.676(0.460, 11.853)	−1.411 (−2.186, −0.635)	0.150(0.017, 0.557)	−6.621 (−7.079, −6.162)	5.358(0.649, 19.544)	−6.355 (−6.950, −5.760)
Female	1.218(0.146, 3.725)	−3.234 (−3.964, −2.504)	1.815(0.221, 5.794)	−2.332 (−3.208, −1.456)	0.050(0.005, 0.185)	−7.863 (−8.415, −7.310)	2.102(0.272, 7.539)	−7.569 (−8.116, −7.022)
XDR-TB	0.143(0.017, 0.436)	7.134 (5.766, 8.503)	0.241(0.030, 0.772)	7.800 (7.022, 8.578)	0.018(0.002, 0.066)	1.762 (0.778, 2.747)	0.611(0.073, 2.111)	41.657 (22.559, 60.755)
Male	0.180(0.022, 0.551)	7.667 (6.590, 8.743)	0.323(0.040, 1.040)	8.287 (7.480, 9.094)	0.028(0.003, 0.102)	2.268 (1.306, 3.229)	0.891(0.111, 3.188)	42.867 (23.321, 62.412)
Female	0.107(0.013, 0.327)	6.460 (5.075, 7.844)	0.159(0.019, 0.509)	6.938 (6.113, 7.763)	0.009(0.001, 0.035)	0.509 (−0.468, 1.486)	0.335(0.039, 1.247)	38.959 (20.485, 57.432)
HIV-DS-TB	1.197(0.991, 1.368)	0.694 (0.426, 0.963)	2.082(1.731, 2.396)	2.062 (1.947, 2.177)	0.098(0.055, 0.169)	1.425 (0.793, 2.056),	5.271(3.261, 8.305)	1.768 (1.236, 2.299)
Male	1.758(1.456, 2.008)	0.948 (0.727, 1.170)	3.053(2.540, 3.520)	1.924 (1.714, 2.134)	0.147(0.084, 0.254)	1.789 (1.167, 2.412)	7.691(4.747, 12.252)	2.061 (1.460, 2.662)
Female	0.616(0.509, 0.702)	0.330 (0.065, 0.596)	1.076(0.894, 1.231)	1.480 (1.344, 1.616)	0.048(0.026, 0.082)	0.804 (0.187, 1.422)	2.727(1.633, 4.284)	0.888 (0.317, 1.459)
HIV-MDR-TB	0.032(0.004, 0.105)	2.222 (1.283, 3.161)	0.109(0.014, 0.368)	4.323 (3.267, 5.379)	0.011(0.001, 0.040)	4.049 (2.830, 5.269)	0.539(0.059, 1.931)	4.081 (2.961, 5.201)
Male	0.047(0.006, 0.154)	1.955 (1.118, 2.792)	0.160(0.020, 0.539)	4.459 (3.457, 5.462)	0.016(0.002, 0.060)	4.381 (3.142, 5.620)	0.789(0.085, 2.848)	4.474 (3.347, 5.601)
Female	0.016(0.002, 0.054)	1.687 (0.702, 2.673)	0.057(0.007, 0.191)	3.890 (2.850, 4.930)	0.005(0.001, 0.020)	3.176 (1.957, 4.395)	0.276(0.031, 0.985)	2.821 (1.699, 3.944)
HIV-XDR-TB	0.003(0.000, 0.010)	13.318 (11.537, 15.099)	0.010(0.001, 0.032)	16.308 (15.468, 17.148)	0.002(0.000, 0.008)	12.035 (10.127, 13.943)	0.097(0.011, 0.353)	67.028 (50.037, 84.019)
Male	0.005(0.001, 0.015)	13.295 (11.538, 15.052)	0.014(0.002, 0.047)	16.412 (15.5424, 17.283)	0.003(0.000, 0.012)	12.062 (9.969, 14.155)	0.141(0.016, 0.525)	64.927 (49.008, 80.845)
Female	0.002(0.000, 0.005)	12.547 (10.996, 14.099)	0.005(0.001, 0.017)	15.497 (14.715, 16.280)	0.001(0.000, 0.004)	10.993 (9.069, 12.917)	0.050(0.006, 0.178)	69.164 (50.560, 87.768)

From 1990 to 2023, the age-standardized burden of HIV-negative TB declined overall. The AAPCs in ASIR, ASPR, ASMR, and ASDR were −3.833, −0.094, −8.183, and −7.501%, respectively. Similar reductions were observed for DS-TB, with corresponding AAPCs of −3.877, −3.369, −8.332, and −7.460%. Nevertheless, Joinpoint analysis identified a recent increase in incidence: between 2019 and 2023, the ASIR increased for both HIV-negative TB (APC = 1.221, 95% CI: 0.536–1.910%) and DS-TB (APC = 1.160, 95% CI: 0.803–1.519%). The ASPR of DS-TB also increased during this period (APC = 1.439, 95% CI: 0.313–2.578%), whereas mortality and DALY rates continued to decline (Table 2).

**Table 2:** Annualized average percent change (AAPC) and annual percent change (APC) for tuberculosis subtypes in China from 1990 to 2023.

Disease	Index	ASIR		ASPR		ASMR		ASDR	
		Years	Change (%, 95CI)	Years	Change (%, 95CI)	Years	Change (%, 95CI)	Years	Change (%, 95CI)
TB	AAPC	1990–2023	−3.833 (−3.995, −3.671)	1990–2023	−0.094 (−0.133, −0.054)	1990–2023	−8.183 (−8.482, −7.885)	1990–2023	−7.501 (−7.782, −7.221)
	APC	1990–1994	−2.042 (−2.511, −1.571)	1990–2000	0.570 (0.524, 0.616)	1990–1992	−5.192 (−7.105, −3.239)	1990–1992	−4.783 (−9.936, 0.664)
	APC	1994–2000	−3.802 (−4.151, −3.453)	2000–2004	−4.904 (−5.109, −4.698)	1992–1997	−8.396 (−9.228, −7.557)	1992–1997	−7.459 (−7.793, −7.125)
	APC	2000–2004	−8.802 (−9.419, −8.181)	2004–2009	2.283 (2.126, 2.439)	1997–2003	−5.456 (−6.106, −4.803)	1997–2003	−6.134 (−6.502, −5.763)
	APC	2004–2011	−2.435 (−2.715, −2.155)	2009–2023	−0.042 (−0.074, −0.010)	2003–2006	−13.734 (−15.475, −11.957)	2003–2006	−11.172 (−13.794, −8.470)
	APC	2011–2019	−6.017 (−6.237, −5.797)			2006–2013	−10.481 (−10.968, −9.992)	2006–2013	−9.116 (−9.510, −8.720)
	APC	2019–2023	1.221 (0.536, 1.910)			2013–2023	−7.038 (−7.332, −6.742)	2013–2023	−6.541 (−7.157, −5.922)
DS-TB	AAPC	1990–2023	−3.877 (−3.967, −3.788)	1990–2023	−3.369 (−3.608, −3.130)	1990–2023	−8.332 (−8.602, −8.062)	1990–2023	−7.460 (−7.680, −7.240)
	APC	1990–2000	−3.718 (−3.791, −3.645)	1990–2000	−2.551 (−2.783, −2.319)	1990–1997	−9.851 (−10.268, −9.433)	1990–1996	−9.130 (−9.538, −8.720)
	APC	2000–2004	−8.492 (−8.816, −8.167)	2000–2004	−8.283 (−9.301, −7.253)	1997–2003	−5.238 (−5.916, −4.557)	1996–2002	−5.715 (−6.251, −5.176)
	APC	2004–2010	−1.598 (−1.784, −1.411)	2004–2011	−0.562 (−1.030, −0.092)	2003–2006	−12.803 (−14.633, −10.934)	2002–2006	−10.234 (−11.185, −9.272)
	APC	2010–2014	−4.399 (−4.737, −4.059)	2011–2019	−6.793 (−7.152, −6.434)	2006–2013	−9.228 (−9.741, −8.712)	2006–2019	−7.744 (−7.907, −7.581)
	APC	2014–2019	−6.854 (−7.087, −6.620)	2019–2023	1.439 (0.313, 2.578)	2013–2023	−7.156 (−7.462, −6.849)	2019–2023	−3.876 (−4.895, −2.846)
	APC	2019–2023	1.160 (0.803, 1.519)						
MDR-TB	AAPC	1990–2023	−2.859 (−3.487, −2.231)	1990–2023	−1.628 (−2.410, −0.846)	1990–2023	−6.714 (−7.265, −6.164)	1990–2023	−6.633 (−7.130, −6.135)
	APC	1990–1991	69.311 (56.950, 82.644)	1990–1993	42.487 (36.868, 48.335)	1990–1992	41.037 (36.941, 45.255)	1990–1992	41.584 (36.960, 46.363)
	APC	1991–1995	11.814 (9.166, 14.527)	1993–2000	−1.314 (−2.977, 0.378)	1992–1994	3.842 (−2.101, 10.145)	1992–1995	1.916 (−1.412, 5.356)
	APC	1995–2000	−4.752 (−6.353, −3.124)	2000–2017	−10.294 (−10.693, −9.894)	1994–2003	−7.207 (−7.705, −6.707)	1995–2003	−7.855 (−8.520, −7.185)
	APC	2000–2016	−11.580 (−11.836, −11.322)	2017–2023	0.503 (−1.635, 2.687)	2003–2009	−17.346 (−18.165, −16.519)	2003–2014	−17.550 (−17.919, −17.180)
	APC	2016–2023	−0.270 (−1.275, 0.746)			2009–2013	−19.568 (−21.054, −18.056)	2014–2023	−5.767 (−6.336, −5.194)
	APC					2013–2023	−6.411 (−6.840, −5.981)		
XDR-TB	AAPC	1990–2023	7.134 (5.766, 8.503)	1990–2023	7.800 (7.022, 8.578)	1990–2023	1.762 (0.778, 2.747)	1990–2023	41.657 (22.559, 60.755)
	APC	1991–1995	54.847 (48.868, 61.067)	1991–1992	111.178 (96.130, 127.377)	1993–1995	76.087 (66.314, 86.433)	1991–1993	695.102 (476.266, 997.039)
	APC	1995–2000	14.012 (9.609, 18.591)	1992–1998	29.191 (27.586, 30.814)	1995–2003	7.135 (5.808, 8.478)	1993–2001	14.461 (6.696, 22.791)
	APC	2000–2006	−2.430 (−5.292, 0.518)	1998–2008	−0.625 (−1.195, −0.051)	2003–2008	−11.106 (−13.348, −8.807)	2001–2014	−11.650 (−14.582, −8.618)
	APC	2006–2016	−7.004 (−8.270, −5.720)	2008–2015	−7.088 (−8.001, −6.166)	2008–2013	−15.037 (−17.180, −12.840)	2014–2023	−2.378 (−7.950, 3.531)
	APC	2016–2023	3.353 (0.949, 5.814)	2015–2019	−1.617 (−3.890, 0.709)	2013–2023	−2.566 (−3.428, −1.696)		
	APC			2019–2023	6.399 (3.941, 8.915)				
HIV-DS-TB	AAPC	1990–2023	0.694 (0.426, 0.963)	1990–2023	2.062 (1.947, 2.177)	1990–2023	1.425 (0.793, 2.056)	1990–2023	1.768 (1.236, 2.299)
	APC	1990–1998	7.209 (6.738, 7.682)	1990–1999	9.758 (9.618, 9.899)	1990–1993	17.091 (13.964, 20.304)	1990–1996	13.466 (12.297, 14.647)
	APC	1998–2009	−1.788 (−2.107, −1.468)	1999–2009	−0.558 (−0.685, −0.430)	1993–2002	10.399 (9.540, 11.266)	1996–2002	9.734 (8.306, 11.180)
	APC	2009–2013	0.447 (−1.074, 1.992)	2009–2015	0.192 (−0.086, 0.471)	2002–2013	−6.340 (−6.878, −5.797)	2002–2012	−5.215 (−5.785, −4.642)
	APC	2013–2023	−1.687 (−2.056, −1.317)	2015–2019	−4.837 (−5.330, −4.341)	2013–2016	4.436 (0.061, 9.003)	2012–2016	1.411 (−1.043, 3.926)
				2019–2023	0.239 (−1.151, 1.648)	2016–2023	−5.645 (−6.240, −5.047)	2016–2023	−4.843 (−5.299, −4.386)
HIV-MDR-TB	AAPC	1990–2023	2.222 (1.283, 3.161)	1990–2023	4.323 (3.267, 5.379)	1990–2023	4.049 (2.830, 5.269)	1990–2023	4.081 (2.961, 5.201)
	APC	1990–1992	77.932 (67.989, 88.463)	1990–1992	87.427 (76.026, 99.567)	1990–1994	59.036 (51.979, 66.421)	1990–1994	57.706 (51.239, 64.449)
	APC	1992–1997	9.299 (6.524, 12.146)	1992–1999	12.043 (10.179, 13.938)	1994–2003	7.587 (5.612, 9.599)	1994–2003	7.983 (6.153, 9.845)
	APC	1997–2002	0.015 (−2.524, 2.621)	1999–2003	−3.282 (−7.045, 0.634)	2003–2012	−15.343 (−16.898, −13.759)	2003–2012	−14.628 (−16.074, −13.155)
	APC	2002–2014	−10.729 (−11.334, −10.120)	2003–2016	−8.207 (−8.809, −7.601)	2012–2023	−2.974 (−4.293, −1.637)	2012–2023	−3.305 (−4.518, −2.077)
	APC	2014–2023	−0.040 (−1.084, 1.015)	2016–2023	0.476 (−1.195, 2.176)				
HIV-XDR-TB	AAPC	1990–2023	13.318 (11.537, 15.099)	1990–2023	16.308 (15.468, 17.148)	1990–2023	12.035 (10.127, 13.943)	1990–2023	67.028 (50.037, 84.019)
	APC	1991–1997	64.165 (58.175, 70.380)	1991–1992	206.915 (187.801, 227.298)	1993–1996	95.316 (81.573, 110.097)	1991–1994	664.307 (520.031, 842.155)
	APC	1997–2004	13.979 (9.821, 18.295)	1992–1998	44.315 (42.755, 45.893)	1996–2003	21.310 (17.627, 25.108)	1994–2002	29.355 (20.346, 39.037)
	APC	2004–2012	−9.965 (−12.655, −7.190)	1998–2004	7.995 (6.828, 9.175)	2003–2012	−9.588 (−11.472, −7.663)	2002–2013	−8.038 (−12.050, −3.844)
	APC	2012–2023	2.096 (0.200, 4.029)	2004–2015	−2.858 (−3.278, −2.436)	2012–2023	1.111 (−0.449, 2.696)	2013–2023	0.556 (−4.491, 5.871)
	APC			2015–2019	2.118 (0.062, 4.215)				
	APC			2019–2023	6.010 (3.877, 8.188)				

Across the major burden indicators, rates were generally higher among males. In 2023, the ASIR of HIV-negative TB was 47.997 per 100,000 in males and 29.015 per 100,000 in females; the corresponding ASMRs were 1.789 and 0.605 per 100,000, and the ASDRs were 77.038 and 31.732 per 100,000, respectively. Similar sex differences were observed for DS-TB and HIV-DS-TB (Table 1 and Supplementary Tables S3–S6).

### MDR-TB and XDR-TB

In 2023, the ASIRs of MDR-TB and XDR-TB were 1.630 (95% UI: 0.196–4.967) and 0.143 (95% UI: 0.017–0.436) per 100,000 population, corresponding to 27,737 and 2,436 incident cases, respectively. MDR-TB also had an ASPR of 2.740, an ASMR of 0.098, and an ASDR of 3.718 per 100,000 population, whereas the corresponding rates for XDR-TB were 0.241, 0.018, and 0.611 per 100,000 population (Table 1 and Supplementary Tables S1–S6).

The long-term trends differed substantially between the two drug-resistant subtypes. From 1990 to 2023, MDR-TB declined across all four indicators, with AAPCs of −2.859% for ASIR, −1.628% for ASPR, −6.714% for ASMR, and −6.633% for ASDR. In contrast, XDR-TB showed positive AAPCs over the full study period, including 7.134% for ASIR, 7.800% for ASPR, 1.762% for ASMR, and 41.657% for ASDR, although its absolute burden remained substantially lower than that of DS-TB and MDR-TB. In the most recent segments, the ASIR of XDR-TB increased from 2016 to 2023 (APC = 3.353, 95% CI: 0.949–5.814%), and its ASPR increased from 2019 to 2023 (APC = 6.399, 95% CI: 3.941–8.915%). By contrast, its ASMR declined from 2013 to 2023 (APC = −2.566, 95% CI: −3.428 to −1.696%), while the recent change in ASDR was not statistically significant (APC = −2.378, 95% CI: −7.950 to 3.531%) (Table 2).

### HIV-associated TB

The absolute burden of HIV-associated TB remained lower than that of HIV-negative TB in 2023. HIV-DS-TB accounted for the largest burden among the HIV-associated subtypes, with an ASIR of 1.197, an ASPR of 2.082, an ASMR of 0.098, and an ASDR of 5.271 per 100,000 population. The corresponding rates were 0.032, 0.109, 0.011, and 0.539 per 100,000 for HIV-MDR-TB and 0.003, 0.010, 0.002, and 0.097 per 100,000 for HIV-XDR-TB (Table 1 and Supplementary Tables S1–S6).

Across the full period from 1990 to 2023, all three HIV-associated TB subtypes showed positive AAPCs across the four burden indicators. The increases were most pronounced for HIV-XDR-TB, with AAPCs of 13.318% for ASIR, 16.308% for ASPR, 12.035% for ASMR, and 67.028% for ASDR. However, recent trends were more heterogeneous. For HIV-DS-TB, the ASIR declined from 2013 to 2023 (APC = −1.687, 95% CI: −2.056 to −1.317%), and both ASMR and ASDR declined from 2016 to 2023. HIV-MDR-TB incidence and prevalence remained broadly stable in their most recent segments, whereas its ASMR and ASDR continued to decline. Conversely, HIV-XDR-TB showed recent increases in ASIR from 2012 to 2023 (APC = 2.096, 95% CI: 0.200–4.029%) and in ASPR from 2019 to 2023 (APC = 6.010, 95% CI: 3.877–8.188%), while recent changes in ASMR and ASDR were not statistically significant (Table 2).

### Projection

For HIV-negative TB, the ASIR was projected to reach 39.692 per 100,000 population (95% PI: 23.504–67.031), corresponding to an AAPC of 0.252% (95% CI: 0.244–0.260%) during 2024–2035. The ASPR was projected to remain broadly stable, whereas the ASMR and ASDR were projected to decline to 0.420 (95% PI: 0.140–1.260) and 28.924 (95% PI: 12.807–65.324) per 100,000 population, respectively. DS-TB showed a similar pattern, with a modest increase in ASIR but continued reductions in mortality and DALY rates. For MDR-TB, incidence and prevalence were projected to increase slightly, whereas the ASMR and ASDR were projected to decline (Table 3).

**Table 3:** Projected burden of tuberculosis and its major subtypes in China, 2024–2035.

Disease	Index	Incidence	Prevalence	Mortality	DALYs
TB	ASR per 100,000 population in 2035 (95% PI)	39.692 (23.504, 67.031)	30649.035 (23645.023, 39727.742)	0.420 (0.140, 1.260)	28.924 (12.807, 65.324)
	AAPC (%), 2024–2035 (95% CI)	0.252 (0.244, 0.260)	−0.015 (−0.016, −0.014)	−7.878 (−8.042, −7.714)	−4.901 (−5.004, −4.797)
DS-TB	ASR per 100,000 population in 2035 (95% PI)	37.933 (19.820, 72.599)	62.523 (32.139, 121.631)	0.327 (0.101, 1.055)	24.935 (10.647, 58.394)
	AAPC (%), 2024–2035 (95% CI)	0.274 (0.268, 0.280)	−0.105 (−0.109, −0.102)	−9.057 (−9.190, −8.923)	−5.480 (−5.562, −5.398)
MDR-TB	ASR per 100,000 population in 2035 (95% PI)	1.717 (0.692, 4.260)	2.818 (1.111, 7.147)	0.060 (0.017, 0.206)	2.636 (0.847, 8.204)
	AAPC (%), 2024–2035 (95% CI)	0.355 (0.328, 0.381)	0.190 (0.176, 0.204)	−3.291 (−3.530, −3.051)	−2.319 (−2.489, −2.149)
XDR-TB	ASR per 100,000 population in 2035 (95% PI)	0.172 (0.077, 0.381)	0.283 (0.111, 0.719)	0.013 (0.003, 0.052)	0.576 (0.007, 44.510)
	AAPC (%), 2024–2035 (95% CI)	1.244 (1.151, 1.336)	1.101 (1.019, 1.183)	−2.447 (−2.626, −2.268)	−0.603 (−0.648, −0.559)
HIV-DS-TB	ASR per 100,000 population in 2035 (95% PI)	1.251 (0.968, 1.618)	2.158 (1.507, 3.091)	0.067 (0.023, 0.193)	3.912 (1.712, 8.937)
	AAPC (%), 2024–2035 (95% CI)	0.304 (0.281, 0.326)	0.247 (0.228, 0.265)	−2.604 (−2.794, −2.413)	−2.013 (−2.160, −1.865)
HIV-MDR-TB	ASR per 100,000 population in 2035 (95% PI)	0.035 (0.011, 0.109)	0.118 (0.035, 0.394)	0.008 (0.002, 0.026)	0.403 (0.139, 1.172)
	AAPC (%), 2024–2035 (95% CI)	0.556 (0.514, 0.597)	0.529 (0.490, 0.569)	−2.368 (−2.541, −2.194)	−1.960 (−2.104, −1.816)
HIV-XDR-TB	ASR per 100,000 population in 2035 (95% PI)	0.003 (0.001, 0.010)	0.012 (0.003, 0.045)	0.002 (0.001, 0.007)	0.097 (0.003, 3.634)
	AAPC (%), 2024–2035 (95% CI)	−0.184 (−0.197, −0.170)	1.443 (1.335, 1.551)	−1.520 (−1.631, −1.408)	−0.132 (−0.142, −0.122)

PIs quantify forecast uncertainty for projected ASRs, whereas AAPC CIs are conditional on the projected point estimates.

For XDR-TB, the ASIR was projected to reach 0.172 per 100,000 population by 2035 (95% PI: 0.077–0.381), with an AAPC of 1.244% (95% CI: 1.151–1.336%). The ASPR was also projected to increase modestly, whereas the ASMR and ASDR showed decreasing point-estimate trends. However, the prediction intervals indicated considerable uncertainty, particularly for the projected XDR-TB DALY rate. These projections should therefore be interpreted cautiously.

Projected trends also varied among the HIV-associated TB subtypes. The ASIRs and ASPRs of HIV-DS-TB and HIV-MDR-TB were projected to increase modestly, whereas their mortality and DALY rates were projected to decline. For HIV-XDR-TB, the ASIR was projected to remain approximately stable, with a small negative AAPC of −0.184% (95% CI: −0.197 to −0.170%), while the ASPR was projected to increase. The ASMR and ASDR of HIV-XDR-TB showed slightly decreasing point-estimate trends. Given the low absolute rates and relatively wide prediction intervals for some HIV-associated drug-resistant TB outcomes, these estimates should also be interpreted cautiously (Table 3).

The projections to 2050 were broadly consistent with those to 2035, with incidence and prevalence generally stabilizing and mortality and DALY rates continuing to decline. The longer projection period was characterized by AAPCs closer to zero and wider prediction intervals. For XDR-TB, the projected ASIR changed only slightly from 0.172 per 100,000 in 2035 to 0.174 per 100,000 in 2050, indicating little change in the point estimate despite greater long-term uncertainty (Supplementary Table S7).

## Discussion

This study provides an updated assessment of TB burden in China and demonstrates heterogeneous epidemiological patterns among different TB subtypes. The principal finding was the heterogeneity in the trajectories of different TB subtypes and burden measures. While the burden of HIV-negative TB, DS-TB, and MDR-TB declined overall during 1990–2023, all HIV-associated TB subtypes showed positive AAPCs over the full study period, although recent trends varied, with HIV-XDR-TB exhibiting the most rapid growth. Despite substantial progress in reducing the overall TB burden, these divergent patterns highlight persistent and emerging challenges for TB control, particularly in relation to XDR-TB and HIV-associated TB. Consequently, the priorities of TB control in China may need to shift from broad population-level interventions toward more targeted strategies focusing on high-risk populations and complex forms of TB.

The widespread implementation of rapid molecular diagnostic technologies, including GeneXpert and whole-genome sequencing (WGS), has substantially improved the detection of drug-resistant tuberculosis and enabled the identification of previously undiagnosed XDR-TB cases (30). XDR-TB demonstrated one of the most unfavorable historical epidemiological trajectories among the TB subtypes. However, the ETS results showed that its projected trends were not uniform across burden indicators: incidence and prevalence were projected to increase modestly through 2035, whereas mortality and DALY rates were projected to decline. In 2023, the ASIR of XDR-TB was relatively low, and was projected to increase modestly to 0.172 per 100,000 (95% PI: 0.077–0.381) by 2035. However, the recorded increase should not be interpreted solely as a true epidemiological rise. Evidence from Guizhou Province showed that the increasing use of molecular drug-susceptibility testing was accompanied by greater detection of rifampicin-resistant TB and shorter diagnostic and treatment delays, suggesting that part of the observed increase may reflect improved surveillance sensitivity (31). Changes in drug-resistance definitions and surveillance coverage over 1990–2023 may also have affected case classification and temporal comparability, while evolving treatment policies could have influenced mortality and DALY trends. The available data do not allow the effects of enhanced detection to be fully separated from changes in underlying transmission, and continued transmission cannot be excluded. The persistence of XDR-TB may reflect multiple factors reported in previous studies, including delayed diagnosis, poor treatment adherence, and limited access to second-line drugs, as well as treatment-related adverse events, financial barriers, and incomplete referral and follow-up across different levels of the healthcare system (32, 33). Without strengthened control measures, drug-resistant TB could undermine the progress made in controlling DS-TB in recent years (34–36). Therefore, expanding access to rapid drug-susceptibility testing, strengthening surveillance of drug resistance, and ensuring timely initiation of individualized treatment should remain central priorities for future TB control strategies. Patient-centered adherence support, adverse-event monitoring, improved referral pathways, contact investigation, and infection-control measures are also required to ensure that improved detection leads to successful treatment and reduced transmission.

In addition, HIV-associated TB showed historically unfavorable trends across the examined subtypes; however, the ETS projections showed heterogeneous patterns across burden indicators. Changes in HIV testing coverage and the integration of TB and HIV services may also have influenced case ascertainment over the study period. Although the absolute burden of HIV-XDR-TB remained relatively low, its projected increase in prevalence warrants attention, while its ASIR was projected to remain approximately 0.003 per 100,000 (95% PI: 0.001–0.010) in 2035. The continuing incidence and prevalence burden of HIV-associated TB may partly reflect the immunocompromised state of people living with HIV, which increases susceptibility to TB and the risk of disease progression. Co-infection with HIV and drug-resistant TB complicates diagnosis and treatment, leading to higher rates of drug interactions and adverse reactions (37, 38). Beyond biological susceptibility, remaining gaps in bidirectional TB/HIV screening and linkage between TB and HIV services, regional differences in diagnostic and treatment capacity, limited uptake of TB preventive treatment, and population mobility may delay diagnosis or interrupt continuity of care (39, 40). This study found that the incidence and prevalence of HIV-DS-TB and HIV-MDR-TB may increase modestly through 2035, whereas their mortality and DALY rates are projected to decline; HIV-XDR-TB incidence is projected to remain broadly stable, despite an increase in prevalence (Table 3). These mixed trends indicate a continuing control challenge rather than a uniform worsening across all indicators (41–44). To further reduce this burden, integrating HIV and TB control services, strengthening early screening, and promoting preventive treatment are crucial steps (41). Timely initiation of antiretroviral and anti-TB treatment and coordinated long-term follow-up should also be strengthened. However, additional quality-improvement support for integrated HIV–TB services did not demonstrate a mortality benefit in one trial (42). Across TB subtypes, the generally higher burden among males may partly reflect differences in smoking, alcohol use, and healthcare-seeking behavior, including delays in seeking care and diagnosis (4). These factors were not directly assessed in this study and remain possible explanations for the observed sex differences.

Future TB control strategies in China should shift from predominantly disease-centered and broadly applied approaches toward more patient-centered, targeted, and risk-stratified prevention and care. Key measures include: curbing the spread of drug-resistant TB through advanced drug sensitivity testing and early intervention; establishing interdisciplinary and interdepartmental collaboration mechanisms to ensure patients receive continuous and comprehensive medical care; leveraging big data and artificial intelligence to provide personalized diagnosis and treatment; and enhancing the service capacity of grassroots healthcare institutions to ensure effective implementation of control measures in low-resource areas (45, 46).

Comparison with other high-burden countries further contextualizes these findings. According to WHO estimates for 2023, India accounted for 26.0% of global incident TB and 27.0% of incident MDR/RR-TB, compared with 6.8 and 7.3%, respectively, for China (3). Thus, although China’s absolute contribution was substantially lower than that of India, it remained one of the leading contributors to the global burden of both TB and drug-resistant TB. South Africa presents a different epidemiological pattern, in which HIV has been a much stronger driver of TB incidence and mortality. Evidence from South Africa indicates that the expansion of antiretroviral therapy and intensified TB screening has contributed substantially to recent declines in TB incidence (47). Compared with these settings, China has achieved marked long-term reductions in DS-TB mortality and DALY rates; however, the persistence of XDR-TB and HIV-associated TB indicates that improvements in conventional TB control have not been sufficient for these smaller but high-risk subgroups.

This study has several limitations. First, the analysis based on GBD 2023 data may be affected by incomplete or inaccurate case reporting, which could compromise the reliability of the data (13, 15). Second, GBD estimates are model-based, and may therefore be affected by uncertainty associated with the underlying data and modeling assumptions (9, 12, 48). Such uncertainty may be greater for rare subtypes, particularly HIV-XDR-TB, for which estimates are based on relatively small case numbers and sparse underlying data. Moreover, national-level estimates may not fully capture the substantial epidemiological and healthcare heterogeneity across provinces and regions of China. Finally, although exponential smoothing methods have previously been applied to tuberculosis surveillance data in China, including forecasts of pulmonary tuberculosis registration rates in Henan Province and epidemic trends in Qinghai Province (49, 50), the ETS model remains a data-driven extrapolation approach that assumes the continuation of historical temporal patterns. It does not explicitly incorporate future changes in TB control policies, diagnostic coverage, treatment availability, healthcare infrastructure, socioeconomic determinants, population mobility, or unexpected public health shocks. As a result, the projections may not fully capture the future dynamics of TB (13).

## Conclusion

This study shows that China has made substantial progress in reducing the overall burden of tuberculosis. However, ETS projections to 2035 indicate divergent trends: incidence and prevalence may increase for XDR-TB and selected HIV-associated TB subtypes, whereas mortality and DALY rates are projected to decline. Projections for HIV-XDR-TB remain subject to substantial uncertainty. These findings suggest that TB control in China is entering a critical stage in which broad population-level strategies should be complemented by more targeted interventions. Future efforts should focus on strengthening surveillance and management of drug-resistant TB, integrating TB and HIV prevention and care, improving early diagnosis, and implementing precise interventions for high-risk populations.

## Data Availability

The datasets presented in this study can be found in online repositories. The names of the repository/repositories and accession number(s) can be found at: http://ghdx.healthdata.org/gbd-results-tool.

## Footnotes

1
https://ghdx.healthdata.org
