# Supplementary material for: Changing epidemiological patterns of tuberculosis in China from 1990 to 2023: trends and future projections of drug-resistant and HIV-associated tuberculosis
Source: Front Public Health. 2026 Sep 18;14:1948171. doi: 10.3389/fpubh.2026.1948171 (PMC13630992; doi:10.3389/fpubh.2026.1948171)
Supplement: Supplementary file 1 [file Supplementary_file_1.docx]

**Supplementary Materials**

**Changing Epidemiological Patterns of Tuberculosis in China from 1990 to 2023: Trends and Future Projections of Drug-Resistant and HIV-Associated Tuberculosis**

**Items**

Table S1: Number of incident tuberculosis cases and subtypes in China from 1990 to 2023

Table S2: Trends in the incidence and prevalence of tuberculosis in China from 1990 to 2023

Table S3: Number of prevalent tuberculosis cases and subtypes in China from 1990 to 2023

Table S4: Number of deaths due to tuberculosis and subtypes in China from 1990 to 2023

Table S5: Trends in the number of tuberculosis deaths and DALYs in China from 1990 to 2023

Table S6: DALYs due to tuberculosis and subtypes in China from 1990 to 2023

Table S7. Exploratory long-term projections of age-standardized tuberculosis burden rates in China through 2050

Methods S1. Detailed ETS forecasting and forecast-period trend analysis

Methods S2. GBD 2023 data extraction and tuberculosis cause classification

**Table S1: Number of** **incident tuberculosis cases and subtypes in China from 1990 to 2023**

| Year | ALL TB | HIV-negative (incidence number) | | | | HIV-positive (incidence number) | | | |
| --- | --- | --- | --- | --- | --- | --- | --- | --- | --- |
|  |  | HIV-negative TB (%,HIV-negative TB /all TB) | DS-TB (%,DS-TB/ HIV-negative TB) | MDR-TB (%,MDR-TB/ HIV-negative TB) | XDR-TB (%,XDR-TB/ HIV-negative TB) | HIV-associated TB (%,HIV-associated TB/ all TB) | HIV-DS-TB (%,HIV-DS-TB/ HIV-associated TB) | HIV-MDR-TB (%,HIV-MDR-TB/ HIV-associated TB) | HIV-XDR-TB (%,HIV-XDR-TB/ HIV-associated TB) |
| 1990 | 1603862 | 1596222(99.524) | 1548087 (96.984) | 48135 (3.016) | 0 (0.000) | 7640(0.476) | 7425(97.186) | 215(2.814) | 0(0) |
| 1991 | 1604060 | 1593950(99.37) | 1510900 (94.789) | 82822 (5.196) | 228 (0.014) | 10110(0.63) | 9633(95.282) | 476(4.708) | 1(0.01) |
| 1992 | 1597844 | 1585233(99.211) | 1470506 (92.765) | 114254 (7.208) | 474 (0.030) | 12611(0.789) | 11878(94.188) | 730(5.789) | 3(0.024) |
| 1993 | 1584739 | 1570627(99.11) | 1429876 (91.038) | 140007 (8.913) | 744 (0.047) | 14112(0.89) | 13145(93.148) | 962(6.817) | 5(0.035) |
| 1994 | 1566634 | 1551081(99.007) | 1392092 (89.748) | 157935 (10.182) | 1055 (0.068) | 15553(0.993) | 14389(92.516) | 1156(7.433) | 8(0.051) |
| 1995 | 1543072 | 1526161(98.904) | 1359000 (89.047) | 165777 (10.862) | 1384 (0.091) | 16911(1.096) | 15600(92.248) | 1299(7.681) | 12(0.071) |
| 1996 | 1512968 | 1494596(98.786) | 1327217 (88.799) | 165617 (11.081) | 1763 (0.118) | 18372(1.214) | 16948(92.249) | 1406(7.653) | 18(0.098) |
| 1997 | 1476201 | 1456178(98.644) | 1292186 (88.739) | 161810 (11.112) | 2183 (0.150) | 20023(1.356) | 18498(92.384) | 1499(7.486) | 26(0.13) |
| 1998 | 1435054 | 1413408(98.492) | 1254967 (88.788) | 155843 (11.026) | 2598 (0.184) | 21646(1.508) | 20032(92.544) | 1580(7.299) | 34(0.157) |
| 1999 | 1392380 | 1369385(98.349) | 1217403 (88.901) | 149039 (10.884) | 2943 (0.215) | 22995(1.651) | 21305(92.651) | 1648(7.167) | 42(0.183) |
| 2000 | 1348995 | 1325168(98.234) | 1179468 (89.005) | 142561 (10.758) | 3138 (0.237) | 23827(1.766) | 22078(92.66) | 1698(7.126) | 51(0.214) |
| 2001 | 1287118 | 1263063(98.131) | 1125467 (89.105) | 134380 (10.639) | 3216 (0.255) | 24055(1.869) | 22283(92.634) | 1711(7.113) | 61(0.254) |
| 2002 | 1199508 | 1175627(98.009) | 1049638 (89.283) | 122740 (10.440) | 3249 (0.276) | 23881(1.991) | 22154(92.768) | 1657(6.939) | 70(0.293) |
| 2003 | 1105500 | 1082003(97.875) | 969189 (89.574) | 109557 (10.125) | 3257 (0.301) | 23497(2.125) | 21865(93.054) | 1556(6.622) | 76(0.323) |
| 2004 | 1026442 | 1003304(97.746) | 903125 (90.013) | 96943 (9.663) | 3236 (0.323) | 23138(2.254) | 21587(93.297) | 1469(6.349) | 82(0.354) |
| 2005 | 982787 | 959857(97.667) | 869789 (90.616) | 86882 (9.052) | 3187 (0.332) | 22930(2.333) | 21470(93.633) | 1375(5.997) | 85(0.371) |
| 2006 | 968471 | 945755(97.654) | 863674 (91.319) | 78979 (8.351) | 3102 (0.328) | 22716(2.346) | 21410(94.251) | 1230(5.415) | 76(0.335) |
| 2007 | 959886 | 937452(97.663) | 863081 (92.065) | 71373 (7.614) | 2998(0.320) | 22434(2.337) | 21258(94.758) | 1105(4.926) | 71(0.316) |
| 2008 | 953948 | 931794(97.678) | 864750 (92.804) | 64179 (6.887) | 2865(0.307) | 22154(2.322) | 21106(95.269) | 980(4.424) | 68(0.307) |
| 2009 | 948246 | 926259(97.681) | 865903 (93.484) | 57632 (6.222) | 2724(0.294) | 21987(2.319) | 21066(95.811) | 859(3.907) | 62(0.282) |
| 2010 | 938585 | 916549(97.652) | 862087 (94.058) | 51873 (5.660) | 2589(0.283) | 22036(2.348) | 21207(96.238) | 774(3.512) | 55(0.25) |
| 2011 | 921223 | 898934(97.58) | 850058 (94.564) | 46437 (5.166) | 2439(0.271) | 22289(2.42) | 21527(96.581) | 713(3.199) | 49(0.22) |
| 2012 | 896286 | 873657(97.475) | 830401 (95.046) | 40995 (4.692) | 2262(0.259) | 22629(2.525) | 21931(96.915) | 655(2.895) | 43(0.19) |
| 2013 | 866595 | 843603(97.347) | 805493 (95.482) | 36023 (4.270) | 2087(0.247) | 22992(2.653) | 22348(97.199) | 603(2.623) | 41(0.178) |
| 2014 | 836328 | 813015(97.212) | 778978 (95.813) | 32082 (3.946) | 1955(0.240) | 23313(2.788) | 22708(97.405) | 563(2.415) | 42(0.18) |
| 2015 | 806862 | 783352(97.086) | 751814 (95.973) | 29641 (3.784) | 1897(0.242) | 23510(2.914) | 22924(97.507) | 541(2.301) | 45(0.191) |
| 2016 | 771363 | 748025(96.974) | 717902 (95.973) | 28226 (3.773) | 1896(0.253) | 23338(3.026) | 22756(97.506) | 532(2.28) | 50(0.214) |
| 2017 | 725509 | 702747(96.863) | 673861 (95.890) | 26983 (3.840) | 1903(0.271) | 22762(3.137) | 22184(97.461) | 526(2.311) | 52(0.228) |
| 2018 | 679937 | 657924(96.762) | 629983 (95.753) | 26019 (3.955) | 1922(0.292) | 22013(3.238) | 21437(97.383) | 523(2.376) | 53(0.241) |
| 2019 | 646025 | 624692(96.698) | 597285 (95.614) | 25454 (4.074) | 1954(0.313) | 21333(3.302) | 20758(97.305) | 522(2.447) | 53(0.248) |
| 2020 | 635294 | 614337(96.701) | 586939 (95.544) | 25395 (4.134) | 2003(0.326) | 20957(3.299) | 20380(97.247) | 524(2.5) | 53(0.253) |
| 2021 | 648976 | 628097(96.783) | 599782 (95.493) | 26180 (4.168) | 2135(0.340) | 20879(3.217) | 20297(97.213) | 529(2.534) | 53(0.254) |
| 2022 | 664704 | 643734(96.845) | 614297 (95.429) | 27135 (4.216) | 2303(0.358) | 20970(3.155) | 20380(97.186) | 536(2.556) | 54(0.258) |
| 2023 | 673420 | 652167(96.844) | 621993 (95.372) | 27737 (4.253) | 2436(0.374) | 21253(3.156) | 20651(97.167) | 549(2.583) | 53(0.249) |

**Table S2: Trends in the incidence and** **prevalence of tuberculosis in China from 1990 to 2023**

| Disease | Incidence (number) | | | Prevalence (number) | | |
| --- | --- | --- | --- | --- | --- | --- |
|  | Incidence number (persons, 95% UI) 2023 | EAPC (95% CI) 1990-2023 | AAPC (95% CI) 1990-2023 | Prevalence number (persons, 95% UI) 2023 | EAPC (95% CI) 1990-2023 | AAPC (95% CI) 1990-2023 |
| TB | 652167(581880,722670) | -3.191(-3.374, -3.008) | -2.604 (-2.795, -2.412) | 494225530(451149870,540923078) | 0.733(0.598,0.869) | 0.832 (0.787, 0.877) |
| Male | 424052(380710,468251) | -2.618(-2.789, -2.446) | -2.210 ( -2.484, -1.937) | 260925118(239045416,284469848) | 0.717(0.585,0.849) | 0.829 (0.785, 0.873) |
| Female | 228115(203278,255519) | -4.067(-4.286, -3.847) | -3.338 (-3.531, -3.145) | 233300412(211757870,256453230) | 0.751(0.611,0.892) | 0.833 (0.783, 0.882) |
| HIV | 31482(15880,50117) | 2.441(0.757,4.153) | 4.348 (3.548 ,5.148) | 547822(292368,1030716) | 7.898(6.600,9.213) | 7.692 (7.530, 7.853) |
| Male | 25139(12875,39746) | 2.966(1.241,4.721) | 4.663 (4.185,5.142) | 415028(217424,778427) | 8.276(6.953,9.616) | 7.987 (7.810, 8.163) |
| Female | 6343(3098,10120) | 0.890(-0.715,2.521) | 2.299 (1.525, 3.073) | 132794(71632,257196) | 6.908(5.659,8.171) | 6.891 (6.710, 7.072) |
| DS-TB | 621993(527769,700178) | -2.994(-3.173, -2.814) | -2.608 -2.797, -2.419) | 1106381(941729,1260801) | -2.383(-2.605, -2.161) | -1.951 (-2.222, -1.681) |
| Male | 404327(345635,455272) | -2.419(-2.584, -2.253) | -2.146 (-2.346, -1.945 | 766126(644512,875420) | -1.922(-2.156, -1.688) | -1.519 (-1.807, -1.231) |
| Female | 217666(182628,247960) | -3.871(-4.084, -3.657) | -3.353 (-3.528, -3.178) | 340256(289193,386195) | -3.236(-3.452, -3.021) | -2.991 (-3.176, -2.805) |
| MDR-TB | 27737(3372,87637) | -5.941(-7.111, -4.757) | -1.591 (-2.174, -1.007) | 47900(6032,157699) | -4.853(-6.073, -3.617) | -0.222 (-1.030, 0.586) |
| Male | 18132(2203,57305) | -5.378(-6.560, -4.181) | -0.901 (-1.619, -0.183), | 33233(4214,109774) | -4.402(-5.646, -3.141) | -0.285 (-0.498, 1.069) |
| Female | 9605(1174,30333) | -6.812(-7.978, -5.630) | -2.221 (-2.957, -1.486) | 14667(1818,47925) | -5.691(-6.882, -4.484) | -1.052 ( -1.897, -0.208) |
| XDR-TB | 2436(296,7697) | 2.442(0.410,4.515) | 7.995 (7.069, 8.921) | 4207(530,13844) | 3.768(1.633,5.948) | 9.466 (8.653, 10.280) |
| Male | 1593(193,5033) | 3.037(0.941,5.175) | 9.219 (8.098, 10.340) | 2918(370,9636) | 4.269(2.084,6.502) | 10.129 (9.326, 10.931) |
| Female | 844(103,2664) | 1.515(-0.441,3.510) | 7.010 (6.025, 7.994) | 1288(160,4208) | 2.836(0.772,4.942) | 8.188 (7.358, 9.017) |
| HIV-DS-TB | 20651(17090,23581) | 1.572(0.953,2.194) | 2.039 (1.776, 2.302) | 35945(29880,41408) | 2.561(1.721,3.408) | 3.180 (2.965, 3.395) |
| Male | 15468(12808,17664) | 1.913(1.290,2.540) | 2.350 (2.104,2.596), | 26861(22333,30975) | 2.890(2.042,3.745) | 3.476 (3.270, 3.682) |
| Female | 5183(4283,5906) | 0.708(0.087,1.334) | 1.473 (1.043 ,1.903) | 9084(7547,10419) | 1.733(0.901,2.571) | 2.492 (2.190,2.795) |
| HIV-MDR-TB | 549(67,1812) | -2.219(-3.858, -0.551) | 3.667 (2.699, 4.635) | 1890(236,6367) | 8.740(5.654,11.917) | 5.790 (4.437, 7.143) |
| Male | 411(50,1354) | -1.868(-3.498, -0.210) | 3.820 (2.750 ,4.890) | 1411(176,4751) | 9.135(6.010,12.352) | 6.118 (4.804, 7.431) |
| Female | 138(17,458) | -3.092(-4.761, -1.394) | 2.865 (1.864,3.866) | 479(60,1616) | 7.765(4.754,10.863) | 5.156 (3.931, 6.381) |
| HIV-XDR-TB | 53(6,175) | 6.798(3.591,10.104) | 14.943 (13.151, 16.735) | 166(21,559) | 8.740(5.654,11.917) | 18.878 (17.535, 20.220) |
| Male | 40(5,132) | 7.331(4.120,10.641) | 15.400 (13.558,17.243) | 124(15,417) | 9.135(6.010,12.352) | 18.350 (17.279, 19.421) |
| Female | 13(2,42) | 5.460(2.243, 8.778) | 13.822 (11.974,15.670) | 42(5,142) | 7.765(4.754,10.863) | 16.806 (16.014, 17.598) |

**Table S3: Number of** **Prevalent tuberculosis cases and subtypes in China from 1990 to 2023**

| Year | ALL TB | HIV-negative (prevalence number) | | | | HIV-positive (prevalence number) | | | |
| --- | --- | --- | --- | --- | --- | --- | --- | --- | --- |
|  |  | HIV-negative TB | DS-TB (%,DS-TB/  HIV-negative TB) | MDR-TB (%,MDR-TB/  HIV-negative TB) | XDR-TB (%,XDR-TB/  HIV-negative TB) | HIV-associated TB (%, HIV-associated TB/ all TB) | HIV-DS-TB (%,HIV-DS-TB/  HIV-associated TB) | HIV-MDR-TB (%,HIV-MDR-TB/  HIV-associated TB) | HIV-XDR-TB (%,HIV-XDR-TB/  HIV-associated TB) |
| 1990 | 377738333 | 377725849(99.997) | 2252765(0.596) | 59085(0.016) | 0(0) | 12484(0.003) | 12090(96.844) | 394(3.156) | 0(0) |
| 1991 | 385801884 | 385787216(99.996) | 2226615(0.577) | 104589(0.027) | 271(0) | 14668(0.004) | 13733(93.626) | 933(6.361) | 2(0.014) |
| 1992 | 393346775 | 393329693(99.996) | 2195836(0.558) | 146762(0.037) | 586(0) | 17082(0.004) | 15606(91.359) | 1471(8.611) | 5(0.029) |
| 1993 | 400522510 | 400502850(99.995) | 2163071(0.54) | 182239(0.046) | 937(0) | 19660(0.005) | 17658(89.817) | 1992(10.132) | 10(0.051) |
| 1994 | 407356337 | 407334046(99.995) | 2134454(0.524) | 207846(0.051) | 1322(0) | 22291(0.005) | 19802(88.834) | 2473(11.094) | 16(0.072) |
| 1995 | 413593483 | 413568564(99.994) | 2111727(0.511) | 220007(0.053) | 1732(0) | 24919(0.006) | 21998(88.278) | 2898(11.63) | 23(0.092) |
| 1996 | 420267071 | 420239080(99.993) | 2093815(0.498) | 222516(0.053) | 2267(0.001) | 27991(0.007) | 24644(88.043) | 3313(11.836) | 34(0.121) |
| 1997 | 427906257 | 427874591(99.993) | 2074852(0.485) | 221937(0.052) | 2942(0.001) | 31666(0.007) | 27867(88.003) | 3749(11.839) | 50(0.158) |
| 1998 | 435764747 | 435729376(99.992) | 2053841(0.471) | 219259(0.05) | 3629(0.001) | 35371(0.008) | 31148(88.061) | 4154(11.744) | 69(0.195) |
| 1999 | 443015807 | 442977323(99.991) | 2030525(0.458) | 215558(0.049) | 4197(0.001) | 38484(0.009) | 33925(88.154) | 4473(11.623) | 86(0.223) |
| 2000 | 448667415 | 448627022(99.991) | 2001729(0.446) | 211614(0.047) | 4502(0.001) | 40393(0.009) | 35647(88.25) | 4647(11.504) | 99(0.245) |
| 2001 | 445523960 | 445482806(99.991) | 1933322(0.434) | 203012(0.046) | 4600(0.001) | 41154(0.009) | 36390(88.424) | 4656(11.314) | 108(0.262) |
| 2002 | 431572340 | 431530906(99.99) | 1814101(0.42) | 187085(0.043) | 4654(0.001) | 41434(0.01) | 36769(88.741) | 4547(10.974) | 118(0.285) |
| 2003 | 413863161 | 413821692(99.99) | 1679377(0.406) | 167697(0.041) | 4682(0.001) | 41469(0.01) | 36970(89.151) | 4371(10.54) | 128(0.309) |
| 2004 | 399753740 | 399712273(99.99) | 1567614(0.392) | 149032(0.037) | 4705(0.001) | 41467(0.01) | 37153(89.597) | 4178(10.075) | 136(0.328) |
| 2005 | 397095007 | 397053400(99.99) | 1517047(0.382) | 135284(0.034) | 4740(0.001) | 41607(0.01) | 37449(90.006) | 4017(9.655) | 141(0.339) |
| 2006 | 406555843 | 406514074(99.99) | 1522494(0.375) | 125858(0.031) | 4768(0.001) | 41769(0.01) | 37795(90.486) | 3832(9.174) | 142(0.34) |
| 2007 | 421100886 | 421059084(99.99) | 1543366(0.367) | 116940(0.028) | 4755(0.001) | 41802(0.01) | 38095(91.132) | 3565(8.528) | 142(0.34) |
| 2008 | 437239523 | 437197709(99.99) | 1569322(0.359) | 108397(0.025) | 4705(0.001) | 41814(0.01) | 38412(91.864) | 3263(7.804) | 139(0.332) |
| 2009 | 451459339 | 451417402(99.991) | 1592376(0.353) | 100291(0.022) | 4633(0.001) | 41937(0.009) | 38824(92.577) | 2976(7.096) | 137(0.327) |
| 2010 | 460166124 | 460123894(99.991) | 1600472(0.348) | 92453(0.02) | 4547(0.001) | 42230(0.009) | 39341(93.159) | 2754(6.521) | 135(0.32) |
| 2011 | 464455003 | 464412325(99.991) | 1585259(0.341) | 84015(0.018) | 4382(0.001) | 42678(0.009) | 39975(93.667) | 2570(6.022) | 133(0.312) |
| 2012 | 468139870 | 468096695(99.991) | 1548832(0.331) | 74734(0.016) | 4108(0.001) | 43175(0.009) | 40669(94.196) | 2376(5.503) | 130(0.301) |
| 2013 | 471501882 | 471458230(99.991) | 1498712(0.318) | 65719(0.014) | 3799(0.001) | 43652(0.009) | 41332(94.685) | 2193(5.024) | 127(0.291) |
| 2014 | 474586664 | 474542636(99.991) | 1445453(0.305) | 58254(0.012) | 3540(0.001) | 44028(0.009) | 41859(95.074) | 2045(4.645) | 124(0.282) |
| 2015 | 477659083 | 477614891(99.991) | 1391818(0.291) | 53348(0.011) | 3399(0.001) | 44192(0.009) | 42114(95.298) | 1954(4.422) | 124(0.281) |
| 2016 | 480670256 | 480626753(99.991) | 1322599(0.275) | 50236(0.01) | 3352(0.001) | 43503(0.009) | 41480(95.35) | 1897(4.361) | 126(0.29) |
| 2017 | 483300440 | 483258632(99.991) | 1227504(0.254) | 47314(0.01) | 3314(0.001) | 41808(0.009) | 39831(95.271) | 1848(4.42) | 129(0.309) |
| 2018 | 485572658 | 485532928(99.992) | 1129345(0.233) | 44884(0.009) | 3296(0.001) | 39730(0.008) | 37789(95.115) | 1809(4.553) | 132(0.332) |
| 2019 | 487604096 | 487566180(99.992) | 1054344(0.216) | 43353(0.009) | 3316(0.001) | 37916(0.008) | 35997(94.939) | 1784(4.705) | 135(0.356) |
| 2020 | 489574365 | 489537349(99.992) | 1024856(0.209) | 42945(0.009) | 3378(0.001) | 37016(0.008) | 35096(94.813) | 1780(4.809) | 140(0.378) |
| 2021 | 491511880 | 491474731(99.992) | 1057480(0.215) | 44800(0.009) | 3653(0.001) | 37149(0.008) | 35191(94.729) | 1810(4.872) | 148(0.398) |
| 2022 | 493158908 | 493121348(99.992) | 1095813(0.222) | 46981(0.01) | 3981(0.001) | 37560(0.008) | 35554(94.659) | 1849(4.923) | 157(0.418) |
| 2023 | 494263531 | 494225530(99.992) | 1106381(0.224) | 47900(0.01) | 4207(0.01) | 38001(0.008) | 35945(94.59) | 1890(4.974) | 166(0.437) |

**Table S4: Number of deaths due to tuberculosis and subtypes in China from 1990 to 2023**

| Year | All TB | HIV-negative (death number) | | | | HIV-positive (death number) | | | |
| --- | --- | --- | --- | --- | --- | --- | --- | --- | --- |
|  |  | HIV-negative TB | DS-TB (%,DS-TB/  HIV-negative TB) | MDR-TB (%,MDR-TB/ HIV-negative TB) | XDR-TB (%,XDR-TB/  HIV-negative TB) | HIV-associated TB (%, HIV-associated TB/ all TB) | HIV-DS-TB (%,HIV-DS-TB/  HIV- associated TB) | HIV-MDR-TB (%,HIV-MDR-TB/ HIV-associated TB) | HIV-XDR-TB (%,HIV-XDR-TB/  HIV-associated TB |
| 1990 | 175304 | 174628(99.614) | 164050(93.943) | 10578(6.057) | 0(0) | 676(0.384) | 626(92.604) | 50(7.396) | 0(0) |
| 1991 | 171501 | 170635(99.495) | 154046(90.278) | 16589(9.722) | 0(0) | 866(0.502) | 764(88.222) | 102(11.778) | 0(0) |
| 1992 | 164701 | 163615(99.341) | 141686(86.597) | 21929(13.403) | 0(0) | 1086(0.655) | 910(83.794) | 176(16.206) | 0(0) |
| 1993 | 156009 | 154683(99.150) | 129224(83.541) | 25317(16.367) | 143(0.092) | 1326(0.843) | 1064(80.241) | 261(19.683) | 1(0.075) |
| 1994 | 146418 | 144840(98.922) | 117763(81.306) | 26775(18.486) | 302(0.209) | 1578(1.066) | 1225(77.63) | 349(22.117) | 4(0.253) |
| 1995 | 137154 | 135296(98.645) | 107839(79.706) | 26999(19.956) | 458(0.339) | 1858(1.337) | 1408(75.78) | 442(23.789) | 8(0.431) |
| 1996 | 127769 | 125638(98.332) | 98824(78.658) | 26220(20.869) | 594(0.473) | 2131(1.64) | 1590(74.613) | 529(24.824) | 12(0.563) |
| 1997 | 119322 | 116916(97.984) | 92244(78.898) | 23991(20.520) | 681(0.582) | 2406(1.977) | 1803(74.938) | 586(24.356) | 17(0.707) |
| 1998 | 113535 | 110816(97.605) | 87689(79.130) | 22363(20.180) | 764(0.689) | 2719(2.339) | 2047(75.285) | 650(23.906) | 22(0.809) |
| 1999 | 109569 | 106489(97.189) | 84407(79.264) | 21233(19.939) | 848(0.796) | 3080(2.734) | 2327(75.552) | 724(23.506) | 29(0.942) |
| 2000 | 106970 | 103476(96.734) | 82153(79.393) | 20389(19.704) | 933(0.902) | 3494(3.163) | 2648(75.787) | 809(23.154) | 37(1.059) |
| 2001 | 103805 | 99900(96.238) | 79290(79.369) | 19598(19.618) | 1012(1.013) | 3905(3.625) | 2961(75.826) | 898(22.996) | 46(1.178) |
| 2002 | 100633 | 96471(95.864) | 76913(79.727) | 18495(19.172) | 1064(1.103) | 4162(3.972) | 3175(76.285) | 933(22.417) | 54(1.297) |
| 2003 | 97459 | 93186(95.616) | 74705(80.168) | 17378(18.649) | 1102(1.183) | 4273(4.2) | 3285(76.878) | 929(21.741) | 59(1.381) |
| 2004 | 92600 | 88102(95.143) | 71275(80.901) | 15736(17.861) | 1091(1.238) | 4498(4.632) | 3498(77.768) | 935(20.787) | 65(1.445) |
| 2005 | 84267 | 79760(94.652) | 65241(81.797) | 13502(16.928) | 1017(1.275) | 4507(5.077) | 3553(78.833) | 887(19.68) | 67(1.487) |
| 2006 | 73753 | 69810(94.654) | 57696(82.647) | 11202(16.046) | 911(1.305) | 3943(5.075) | 3147(79.812) | 736(18.666) | 60(1.522) |
| 2007 | 66452 | 62866(94.604) | 52315(83.217) | 9703(15.434) | 847(1.347) | 3586(5.12) | 2885(80.452) | 645(17.987) | 56(1.562) |
| 2008 | 62045 | 58540(94.351) | 49465(84.498) | 8300(14.178) | 775(1.324) | 3505(5.347) | 2870(81.883) | 581(16.576) | 54(1.541) |
| 2009 | 58306 | 55038(94.395) | 47272(85.890) | 7064(12.835) | 702(1.275) | 3268(5.307) | 2727(83.446) | 492(15.055) | 49(1.499) |
| 2010 | 53573 | 50549(94.355) | 44062(87.167) | 5868(11.609) | 619(1.225) | 3024(5.343) | 2567(84.888) | 413(13.657) | 44(1.455) |
| 2011 | 48627 | 45855(94.299) | 40533(88.394) | 4788(10.442) | 535(1.167) | 2772(5.393) | 2393(86.328) | 341(12.302) | 38(1.371) |
| 2012 | 45124 | 42630(94.473) | 38143(89.475) | 4014(9.416) | 473(1.110) | 2494(5.238) | 2184(87.57) | 277(11.107) | 33(1.323) |
| 2013 | 41253 | 38848(94.170) | 35078(90.296) | 3354(8.634) | 416(1.071) | 2405(5.509) | 2130(88.565) | 245(10.187) | 30(1.247) |
| 2014 | 39485 | 36902(93.458) | 33554(90.927) | 2962(8.027) | 386(1.046) | 2583(6.14) | 2307(89.315) | 244(9.446) | 32(1.239) |
| 2015 | 38633 | 35857(92.814) | 32652(91.062) | 2821(7.867) | 385(1.074) | 2776(6.704) | 2484(89.481) | 257(9.258) | 35(1.261) |
| 2016 | 38092 | 35192(92.387) | 32004(90.941) | 2790(7.928) | 398(1.131) | 2900(7.075) | 2590(89.31) | 271(9.345) | 39(1.345) |
| 2017 | 36300 | 33446(92.138) | 30360(90.773) | 2686(8.031) | 400(1.196) | 2854(7.289) | 2545(89.173) | 269(9.425) | 40(1.402) |
| 2018 | 34431 | 31687(92.030) | 28719(90.633) | 2569(8.107) | 399(1.259) | 2744(7.381) | 2442(88.994) | 261(9.512) | 41(1.494) |
| 2019 | 32391 | 29805(92.016) | 26966(90.475) | 2444(8.200) | 395(1.325) | 2586(7.393) | 2298(88.863) | 248(9.59) | 40(1.547) |
| 2020 | 30841 | 28399(92.082) | 25655(90.338) | 2349(8.271) | 395(1.391) | 2442(7.337) | 2166(88.698) | 236(9.664) | 40(1.638) |
| 2021 | 30090 | 27769(92.286) | 25057(90.234) | 2310(8.319) | 403(1.451) | 2321(7.161) | 2056(88.583) | 226(9.737) | 39(1.68) |
| 2022 | 29898 | 27662(92.521) | 24934(90.138) | 2309(8.347) | 418(1.511) | 2236(7.479) | 1977(88.417) | 219(9.794) | 40(1.789) |
| 2023 | 26762 | 24752(92.489) | 22291(90.057) | 2073(8.375) | 388(1.568) | 2010(7.511) | 1775(88.308) | 198(9.851) | 37(1.841) |

**Table S5: Trends in the number of tuberculosis deaths and DALYs in China from 1990 to 2023**

| Disease | Death (number) | | | DALY (number) | | |
| --- | --- | --- | --- | --- | --- | --- |
|  | Death number (persons, 95% UI) 2023 | EAPC (95% CI) 1990-2023 | AAPC (95% CI) 1990-2023 | DALY number (persons, 95% UI) 2023 | EAPC (95% CI) 1990-2023 | AAPC (95% CI) 1990-2023 |
| TB | 24752(18360,34622) | -6.117(-6.333, -5.901) | -5.848 (-6.181, -5.516) | 990704(779739,1281147) | -6.341(-6.498, -6.184) | -5.857 (-6.213, -5.500) |
| Male | 18216(13344,25965) | -5.564(-5.773, -5.355) | -5.132 (-5.430, -4.835) | 720400(565754,937267) | -5.764(-5.912, -5.616) | -5.449 (-5.820, -5.078) |
| Female | 6536(4118,13220) | -7.345(-7.618, -7.072) | -6.877 (-7.214, -6.541) | 270305(197522,444713) | -7.549(-7.752, -7.344) | -6.954 (-7.254, -6.653) |
| HIV | 30128(20481,40124) | 8.782(7.653,9.923) | 9.746 (8.814, 10.678) | 1294431(885614,1742837) | 8.289(7.140,9.451) | 9.293 (8.646, 9.940) |
| Male | 23380(15926,30872) | 9.419(8.307,10.544) | 10.270 (9.347, 11.192) | 998428(689059,1363858) | 8.981(7.833,10.142) | 9.569 (8.896,10.241) |
| Female | 6748(4665,9409) | 7.147(5.946,8.361) | 8.456 (7.490, 9.423) | 296003(201773,421251) | 6.615(5.425,7.818) | 7.675 (6.841, 8.509) |
| DS-TB | 22291(15036,32595) | -5.793(-5.979, -5.606) | -5.802 (-6.086, -5.518) | 910233(665043,1202986) | -6.004(-6.146, -5.861) | -5.824 (-6.067, -5.581) |
| Male | 16401(10866,24327) | -5.239(-5.413, -5.066) | -5.322 (-5.683, -4.961), | 661233(495392,861630) | -5.427(-5.556, -5.299) | -5.356 (-5.632, -5.081) |
| Female | 5889(3320,11820) | -7.023(-7.267, -6.778) | -6.689 (-6.938, -6.441), | 249000(170282,415193) | -7.207(-7.389, -7.024) | -6.923 (-7.151, -6.695) |
| MDR-TB | 2073(234,7476) | -8.453(-9.587, -7.306) | -4.631 (-5.071, -4.191) | 69006(8519,243083) | -9.058(-10.160, -7.942) | -5.152 (-5.729, -4.575) |
| Male | 1528(172,5683) | -7.902(-9.051, -6.738) | -3.908 (-4.532, -3.283) | 50666(6103,185714) | -8.461(-9.576, -7.332) | -4.412 (-4.993, -3.831) |
| Female | 544(56,2009) | -9.681(-10.809, -8.539) | -5.363 (-5.900, -4.826) | 18340(2379,67663) | -10.327(-11.429, -9.212) | -6.078 (-6.575, -5.581) |
| XDR-TB | 388(44,1408) | -1.481(-3.338,0.411) | 4.458 (3.473,5.444) | 11466(1361,39819) | 3.156(-1.661,8.208) | 43.194 (24.521, 61.868) |
| Male | 286(33,1037) | -0.909(-2.808,1.028) | 5.000 (4.025, 5.975) | 8500(1040,30414) | 3.903(-0.992,9.040) | 44.916 (25.538,64.294) |
| Female | 102(11,389) | -2.785(-4.579, -0.958) | 3.185 (2.258, 4.112) | 2965(341,10623) | 1.554(-3.145,6.480) | 40.227 (22.364, 58.090) |
| HIV-DS-TB | 1775(998,3049) | 2.757(1.420,4.112) | 3.397 (2.703, 4.091) | 86778(54353,137853) | 1.936(0.614,3.275) | 2.93.648 (3.303, 3.992) |
| Male | 1359(770,2338) | 2.202(0.861,3.562) | 3.864 (3.180, 4.547) | 65806(41099,104987) | 2.513(1.183,3.859) | 3.648 (3.303, 3.992) |
| Female | 416(222,713) | 0.822(-0.557,2.221) | 1.860 (1.135, 2.586) | 20972(12805,32838) | 0.578(-0.755,1.930) | 1.569 (0.965, 2.174) |
| HIV-MDR-TB | 198(22,723) | -0.843(-3.249,1.623) | 5.443 (3.979, 6.907) | 8859(968,31580) | -1.129(-3.531,1.332) | 5.389 (4.170, 6.608) |
| Male | 152(16,552) | -0.304(-2.707,2.158) | 5.722 (4.495,6.949) | 6752(726,24176) | -0.547(-2.958,1.923) | 5.871 (4.657, 7.084) |
| Female | 46(5,168) | -2.179(-4.614,0.319) | 4.355 (2.866, 5.844) | 2107(239,7545) | -2.492(-4.898, -0.024) | 4.251 (3.007,5.495) |
| HIV-XDR-TB | 37(4,140) | 5.043(1.993,8.185) | 14.108 (11.880,16.335) | 1583(182,5841) | 11.838(5.372,18.700) | 69.833 (50.995, 88.672) |
| Male | 28(3,108) | 5.642(2.575,8.802) | 14.651 (12.444,16.859) | 1207(136,4525) | 12.480(6.002,19.354) | 70.119 (51.855, 88.382) |
| Female | 9(1,31) | 3.541(0.505,6.669) | 12.213 (9.983, 14.443) | 376(45,1334) | 10.314(3.861,17.167) | 70.240 (51.566, 88.914) |

**Table S6:** **DALYs due to tuberculosis and subtypes in China from 1990 to 2023**

| Year | ALL TB | HIV-negative (DALYs number) | | | | HIV-positive (DALYs number) | | | |
| --- | --- | --- | --- | --- | --- | --- | --- | --- | --- |
|  |  | HIV-negative TB | DS-TB (%,DS-TB/  HIV-negative TB) | MDR-TB (%,MDR-TB/  HIV-negative TB) | XDR-TB (%,XDR-TB/  HIV-negative TB) | HIV-associated TB (%, HIV-associated TB/ all TB) | HIV-DS-TB (%,HIV-DS-TB/  HIV-associated TB) | HIV-MDR-TB (%,HIV-MDR-TB/  HIV-associated TB) | HIV-XDR-TB (%,HIV-XDR-TB/  HIV-associated TB |
| 1990 | 7448982 | 7412819(99.515) | 6986303(94.246) | 426516(5.754) | 0(0) | 36163(0.485) | 33712(93.222) | 2451(6.778) | 0(0) |
| 1991 | 7264254 | 7218776(99.374) | 6551246(90.753) | 667440(9.246) | 90(0.001) | 45478(0.626) | 40439(88.92) | 5038(11.078) | 1(0.002) |
| 1992 | 6958421 | 6902285(99.193) | 6024907(87.289) | 877183(12.709) | 195(0.003) | 56136(0.807) | 47580(84.758) | 8554(15.238) | 2(0.004) |
| 1993 | 6580891 | 6513029(98.969) | 5500006(84.446) | 1007354(15.467) | 5669(0.087) | 67862(1.031) | 55202(81.344) | 12589(18.551) | 71(0.105) |
| 1994 | 6181971 | 6101644(98.701) | 5028168(82.407) | 1061772(17.401) | 11703(0.192) | 80327(1.299) | 63357(78.874) | 16785(20.896) | 185(0.23) |
| 1995 | 5795863 | 5701684(98.375) | 4619138(81.014) | 1065051(18.68) | 17495(0.307) | 94179(1.625) | 72630(77.119) | 21200(22.51) | 349(0.371) |
| 1996 | 5399832 | 5291979(98.003) | 4242855(80.175) | 1026648(19.4) | 22476(0.425) | 107853(1.997) | 81953(75.986) | 25342(23.497) | 558(0.517) |
| 1997 | 5035672 | 4913945(97.583) | 3955845(80.502) | 932565(18.978) | 25536(0.52) | 121727(2.417) | 92860(76.285) | 28092(23.078) | 775(0.637) |
| 1998 | 4774602 | 4637200(97.122) | 3746673(80.796) | 862153(18.592) | 28374(0.612) | 137402(2.878) | 105239(76.592) | 31129(22.655) | 1034(0.753) |
| 1999 | 4577802 | 4422926(96.617) | 3582857(81.006) | 808978(18.291) | 31091(0.703) | 154876(3.383) | 118916(76.781) | 34615(22.35) | 1345(0.868) |
| 2000 | 4430162 | 4255862(96.066) | 3454901(81.18) | 767309(18.029) | 33651(0.791) | 174300(3.934) | 134094(76.933) | 38491(22.083) | 1715(0.984) |
| 2001 | 4250453 | 4057965(95.471) | 3295432(81.209) | 726686(17.908) | 35847(0.883) | 192488(4.529) | 148005(76.891) | 42351(22.002) | 2132(1.108) |
| 2002 | 4058767 | 3854581(94.969) | 3142229(81.519) | 675267(17.519) | 37085(0.962) | 204186(5.031) | 157846(77.305) | 43876(21.488) | 2464(1.207) |
| 2003 | 3860135 | 3649454(94.542) | 2988779(81.897) | 622885(17.068) | 37790(1.035) | 210681(5.458) | 164003(77.844) | 43953(20.862) | 2725(1.293) |
| 2004 | 3620003 | 3399619(93.912) | 2807021(82.569) | 555688(16.346) | 36909(1.086) | 220384(6.088) | 173372(78.668) | 44023(19.976) | 2989(1.356) |
| 2005 | 3298759 | 3079518(93.354) | 2571032(83.488) | 474331(15.403) | 34154(1.109) | 219241(6.646) | 174712(79.689) | 41473(18.917) | 3056(1.394) |
| 2006 | 2945693 | 2749941(93.355) | 2322677(84.463) | 396597(14.422) | 30667(1.115) | 195752(6.645) | 158033(80.731) | 34947(17.853) | 2772(1.416) |
| 2007 | 2692384 | 2511115(93.267) | 2139302(85.193) | 343418(13.676) | 28394(1.131) | 181269(6.733) | 147623(81.439) | 31008(17.106) | 2638(1.455) |
| 2008 | 2532111 | 2355689(93.033) | 2036421(86.447) | 293465(12.458) | 25803(1.095) | 176422(6.967) | 146120(82.824) | 27778(15.745) | 2524(1.431) |
| 2009 | 2392887 | 2226840(93.061) | 1953961(87.746) | 249641(11.211) | 23237(1.043) | 166047(6.939) | 140091(84.368) | 23669(14.254) | 2287(1.377) |
| 2010 | 2215637 | 2060639(93.004) | 1832763(88.941) | 207533(10.071) | 20342(0.987) | 154998(6.996) | 132978(85.793) | 19977(12.889) | 2043(1.318) |
| 2011 | 2032142 | 1889633(92.987) | 1701957(90.068) | 170166(9.005) | 17510(0.927) | 142509(7.013) | 124241(87.181) | 16491(11.572) | 1777(1.247) |
| 2012 | 1889348 | 1759666(93.136) | 1601806(91.029) | 142472(8.097) | 15388(0.874) | 129682(6.864) | 114687(88.437) | 13470(10.387) | 1525(1.176) |
| 2013 | 1743938 | 1618636(92.815) | 1485848(91.796) | 119301(7.37) | 13487(0.833) | 125302(7.185) | 112005(89.388) | 11883(9.483) | 1414(1.128) |
| 2014 | 1658451 | 1527832(92.124) | 1411186(92.365) | 104285(6.826) | 12361(0.809) | 130619(7.876) | 117645(90.067) | 11531(8.828) | 1443(1.105) |
| 2015 | 1602108 | 1465974(91.503) | 1356189(92.511) | 97639(6.66) | 12146(0.829) | 136134(8.497) | 122787(90.196) | 11796(8.665) | 1551(1.139) |
| 2016 | 1548969 | 1410101(91.035) | 1303113(92.413) | 94649(6.712) | 12339(0.875) | 138868(8.965) | 125064(90.06) | 12132(8.736) | 1672(1.204) |
| 2017 | 1454700 | 1319711(90.72) | 1217578(92.261) | 89894(6.812) | 12238(0.927) | 134989(9.28) | 121353(89.898) | 11920(8.83) | 1716(1.271) |
| 2018 | 1358698 | 1229860(90.518) | 1132769(92.106) | 85025(6.913) | 12067(0.981) | 128838(9.482) | 115620(89.741) | 11494(8.921) | 1724(1.338) |
| 2019 | 1266960 | 1145614(90.422) | 1053464(91.956) | 80312(7.01) | 11837(1.033) | 121346(9.578) | 108725(89.599) | 10918(8.997) | 1703(1.403) |
| 2020 | 1202059 | 1087241(90.448) | 998847(91.87) | 76697(7.054) | 11697(1.076) | 114818(9.552) | 102732(89.474) | 10402(9.06) | 1684(1.467) |
| 2021 | 1177151 | 1067621(90.695) | 980629(91.852) | 75169(7.041) | 11823(1.107) | 109530(9.305) | 97903(89.385) | 9957(9.091) | 1670(1.525) |
| 2022 | 1174438 | 1068106(90.946) | 980686(91.815) | 75199(7.04) | 12220(1.144) | 106332(9.054) | 94970(89.315) | 9682(9.105) | 1680(1.58) |
| 2023 | 1087924 | 990704(91.064) | 910233(91.877) | 69006(6.965) | 11466(1.157) | 97220(8.936) | 86778(89.259) | 8859(9.112) | 1583(1.628) |

**Table S7. Exploratory long-term projections of age-standardized tuberculosis burden rates in China through 2050**

| **Disease** | **Metric** | **Incidence** | **Prevalence** | **Mortality** | **DALYs** |
| --- | --- | --- | --- | --- | --- |
| **TB** | ASR per 100,000 in 2050 (95% PI) | 40.095 (12.791, 125.687) | 30644.350 (19143.028, 49055.782) | 0.257 (0.019, 3.553) | 21.423 (3.060, 149.958) |
|  | AAPC (%), 2024–2050 (95% CI) | 0.145 (0.142, 0.147) | -0.007 (-0.007, -0.007) | -5.202 (-5.253, -5.152) | -3.219 (-3.250, -3.187) |
| **DS-TB** | ASR per 100,000 in 2050 (95% PI) | 38.558 (8.180, 181.747) | 62.258 (14.590, 265.677) | 0.156 (0.008, 3.167) | 16.086 (1.804, 143.448) |
|  | AAPC (%), 2024–2050 (95% CI) | 0.178 (0.177, 0.180) | -0.061 (-0.061, -0.060) | -6.622 (-6.668, -6.576) | -3.986 (-4.014, -3.958) |
| **MDR-TB** | ASR per 100,000 in 2050 (95% PI) | 1.723 (0.331, 8.961) | 2.823 (0.522, 15.279) | 0.058 (0.006, 0.544) | 2.572 (0.328, 20.173) |
|  | AAPC (%), 2024–2050 (95% CI) | 0.164 (0.158, 0.170) | 0.088 (0.085, 0.091) | -1.535 (-1.593, -1.477) | -1.079 (-1.120, -1.038) |
| **XDR-TB** | ASR per 100,000 in 2050 (95% PI) | 0.174 (0.041, 0.738) | 0.286 (0.052, 1.556) | 0.012 (0.001, 0.158) | 0.572 (0.001, 1385.078) |
|  | AAPC (%), 2024–2050 (95% CI) | 0.573 (0.551, 0.595) | 0.508 (0.488, 0.527) | -1.139 (-1.182, -1.096) | -0.279 (-0.290, -0.269) |
| **HIV-DS-TB** | ASR per 100,000 in 2050 (95% PI) | 1.255 (0.788, 2.001) | 2.164 (1.128, 4.151) | 0.065 (0.009, 0.447) | 3.830 (0.856, 17.143) |
|  | AAPC (%), 2024–2050 (95% CI) | 0.140 (0.135, 0.146) | 0.114 (0.110, 0.118) | -1.212 (-1.258, -1.167) | -0.936 (-0.971, -0.900) |
| **HIV-MDR-TB** | ASR per 100,000 in 2050 (95% PI) | 0.035 (0.004, 0.280) | 0.119 (0.013, 1.055) | 0.008 (0.001, 0.070) | 0.395 (0.057, 2.736) |
|  | AAPC (%), 2024–2050 (95% CI) | 0.256 (0.247, 0.266) | 0.244 (0.235, 0.254) | -1.102 (-1.144, -1.060) | -0.911 (-0.946, -0.876) |
| **HIV-XDR-TB** | ASR per 100,000 in 2050 (95% PI) | 0.003 (0.001, 0.027) | 0.012 (0.001, 0.136) | 0.002 (0.001, 0.022) | 0.097 (0.001, 52.463) |
|  | AAPC (%), 2024–2050 (95% CI) | -0.085 (-0.088, -0.082) | 0.665 (0.639, 0.690) | -0.705 (-0.732, -0.679) | -0.061 (-0.063, -0.059) |

**Supplementary Methods S1. Detailed ETS forecasting and forecast-period trend analysis**

**S1.1 Model specification**

Let $y_{t}$ denote the observed annual age-standardized rate (ASR) in year $t$. Each series was modeled on the natural logarithmic scale, $z_{t}=ln\left( y_{t} \right)$, to stabilize variance and ensure positive back-transformed forecasts. Leading observations with non-positive ASRs in selected very-low-burden series were excluded before logarithmic transformation.

A damped-trend exponential smoothing model with an ETS(A,Ad,N) specification was fitted separately to each combination of TB subtype and burden measure. The model included additive errors, a damped additive trend, and no seasonal component because the data were annual. The one-step-ahead prediction and state-updating equations were:

$$\hat{z}_{t\mid t-1}=\mathcal{l}_{t-1}+\phi b_{t-1}$$

$$\mathcal{l}_{t}=\alpha z_{t}+\left( 1-\alpha\right)\left( \mathcal{l}_{t-1}+\phi b_{t-1} \right)$$

$$b_{t}=\beta\left( \mathcal{l}_{t}-\mathcal{l}_{t-1} \right)+\left( 1-\beta\right)\phi b_{t-1}$$

where $\mathcal{l}_{t}$ is the local level, $b_{t}$ is the local trend, $\alpha$ and $\beta$ are smoothing parameters, and $\phi$ is the damping parameter. The $h$-step-ahead forecast on the logarithmic scale was calculated as:

$$\hat{z}_{T+h\mid T}=\mathcal{l}_{T}+b_{T}\sum_{j=1}^{h} \phi^{j}$$

The smoothing parameters and initial states were jointly estimated by minimizing the sum of squared one-step-ahead prediction errors. To constrain the trend-smoothing parameter, $\beta$ was parameterized as $\beta=\alpha r$, where $0<\alpha<1$ and $0<r<1$. Parameter estimation used bounded L-BFGS-B optimization with multiple starting values, and the solution with the lowest residual sum of squares was retained.

**S1.2 Selection of the damping parameter**

The damping parameter was selected separately for each series from candidate values of 0.80, 0.85, 0.90, 0.93, and 0.95 using rolling-origin validation [25]. Six training endpoints from 2013 to 2018 were used. At each origin, the model was fitted using observations available up to that year and evaluated against the subsequent five annual observations.

For each candidate value of $\phi$, pooled validation error was calculated on the logarithmic scale as:

$$\text{RMSE}_{\phi}=\sqrt{\frac{1}{30}\sum_{o=1}^{6} \sum_{h=1}^{5} \left( z_{o+h}-\hat{z}_{o+h\mid o,\phi} \right)^{2}}$$

The candidate value with the lowest pooled log-scale RMSE was retained. The selected model was then refitted using all available positive observations through 2023.

**S1.3 Prediction intervals and back-transformation**

For forecast horizon $h$, the forecast-variance factor was calculated as $q_{h}=1+\sum_{j=1}^{h-1} \left( \alpha+\beta\sum_{i=1}^{j} \phi^{i} \right)^{2}$. The residual variance was estimated as $\hat{\sigma}^{2}=\text{SSE}/\left( n-5 \right)$, where $n$ is the number of observations used to fit the model.

The 95% prediction interval on the logarithmic scale was calculated as [26]:

$$\hat{z}_{T+h\mid T}\pm1.96\hat{\sigma}\sqrt{q_{h}}$$

Point forecasts and prediction limits were subsequently back-transformed using the exponential function. For tabular presentation, lower prediction limits below 0.001 per 100,000 population were displayed as 0.001.

A single fitted trajectory was used to generate projections from 2024 to 2050. The year 2035 was designated as the primary projection endpoint, whereas projections to 2050 were treated as exploratory.

**S1.4 Forecast-period APCs and AAPCs**

Forecast-period trends were estimated from the annual projected point estimates. For a projection period ending in year $H$, segmented log-linear regression was fitted as:

$$\log\left( \hat{y}_{t} \right)=\gamma_{0}+\gamma_{1}\left( t-2024 \right)+\sum_{j=1}^{m} \delta_{j}\max\left( 0,t-\tau_{j} \right)+\varepsilon_{t}$$

where $\tau_{j}$ denotes a candidate joinpoint. A maximum of one joinpoint was allowed for 2024-2035 and three joinpoints for 2024-2050, with at least five years between adjacent segment boundaries. Candidate joinpoint years were evaluated by grid search, and the model with the lowest Bayesian information criterion was selected. The Bayesian information criterion was defined as $\text{BIC}=n\ln\left( \text{SSE}/n \right)+p\ln\left( n \right)$, where $p$ is the number of regression parameters.

The mean annual log-scale slope over the projection period was calculated as:

$$\bar{s}=\gamma_{1}+\sum_{j=1}^{m} \delta_{j}\frac{H-\tau_{j}}{H-2024}$$

The forecast-period AAPC was then obtained as:

$$\text{AAPC}=100\left[ \exp\left( \bar{s} \right)-1 \right]$$

Segment-specific APCs were calculated from the corresponding segment slopes using the same exponential transformation. The 95% confidence intervals for APCs and AAPCs were derived from the covariance matrix of the segmented regression using the appropriate $t$-distribution critical value. These confidence intervals were conditional on the projected point trajectory and therefore did not incorporate the full uncertainty represented by the ETS prediction intervals.

**S1.5 Sensitivity analysis and software implementation**

Because the XDR-TB DALY series showed greater variability and particularly wide long-term prediction intervals, sensitivity analyses were conducted using alternative historical windows beginning in 1999, 2004, and 2009. The damping parameter and all smoothing parameters were re-estimated independently for each window using the same rolling-origin validation procedure. The model fitted to the 2009-2023 window produced smoothing-parameter estimate at the boundary of the permitted range, warranting caution when interpreting forecasts from this shorter observation window.

Forecast generation and forecast-period trend analyses were implemented using custom Python scripts in Python version 3.12.13 with NumPy version 2.3.5, pandas version 2.2.3, and SciPy version 1.17.0.

**Methods S2. GBD 2023 data extraction and tuberculosis cause classification**

**S2.1. GBD 2023 data extraction**

Historical tuberculosis burden estimates for China were extracted from the Global Burden of Disease Study 2023 (GBD 2023) Results Tool. Data were retrieved for China (location ID 6) for each year from 1990 through 2023. The downloaded datasets contained estimates stratified by year, sex, age, cause, measure, and metric, together with the corresponding lower and upper bounds of the 95% uncertainty interval (UI).

The extracted measures were deaths (measure ID 1), disability-adjusted life years (DALYs; measure ID 2), prevalence (measure ID 5), and incidence (measure ID 6). Both numbers (metric ID 1) and rates (metric ID 3) were obtained. Sex-specific estimates were available for males (sex ID 1), females (sex ID 2), and both sexes combined (sex ID 3). The downloaded age categories included age-specific groups as well as all ages (age ID 22) and age-standardized estimates (age ID 27). Age-standardized rates were used for comparisons of temporal trends, whereas numbers and age-specific rates were used where appropriate for descriptive and subgroup analyses.

The underlying historical burden estimates, including point estimates and 95% UIs, were obtained from the GBD 2023 data exports. Quantities generated as part of the present analysis, including subtype proportions, APC and AAPC estimates from Joinpoint regression, and ETS-based projections, were subsequently calculated from these extracted GBD estimates and were not direct outputs of the GBD Results Tool.

**S2.2. GBD 2023 cause and subcause identifiers**

The tuberculosis-related causes and subcauses used in this study were identified according to the cause identifiers contained in the original GBD 2023 data exports.

| **Cause ID** | **GBD 2023 cause/subcause** | **Terminology used in this study** |
| --- | --- | --- |
| 297 | Tuberculosis | TB / HIV-negative TB parent category |
| 934 | Drug-susceptible tuberculosis | DS-TB |
| 946 | Multidrug-resistant tuberculosis without extensive drug resistance | MDR-TB |
| 947 | Extensively drug-resistant tuberculosis | XDR-TB |
| 948 | HIV/AIDS – Drug-susceptible Tuberculosis | HIV-DS-TB |
| 949 | HIV/AIDS – Multidrug-resistant Tuberculosis without extensive drug resistance | HIV-MDR-TB |
| 950 | HIV/AIDS – Extensively drug-resistant Tuberculosis | HIV-XDR-TB |
| 954 | Latent tuberculosis infection | LTBI |

For conciseness, “MDR-TB” and “HIV-MDR-TB” in the manuscript refer specifically to the GBD 2023 categories multidrug-resistant tuberculosis without extensive drug resistance and HIV/AIDS – multidrug-resistant tuberculosis without extensive drug resistance, respectively. Parent HIV/AIDS causes that were also present in the downloaded data were not treated as TB subtype outcomes in the present study.

**S2.3. Tuberculosis classification by HIV and drug-resistance status**

Disease categories were retained according to the case definitions and modelling hierarchy used in GBD 2023 rather than being retrospectively reclassified according to subsequently updated clinical definitions.

In the GBD 2023 tuberculosis framework, drug-susceptible TB is defined as TB susceptible to isoniazid and rifampicin. MDR-TB without extensive drug resistance represents TB resistant to at least isoniazid and rifampicin but not meeting the GBD 2023 definition of XDR-TB. XDR-TB is defined as TB resistant to isoniazid and rifampicin, plus any fluoroquinolone and at least one second-line injectable drug (amikacin, kanamycin, or capreomycin). The corresponding drug-resistance definitions are applied to HIV-associated TB.

GBD 2023 first separates HIV-associated TB from TB among HIV-negative individuals and subsequently partitions each stratum according to drug-resistance status. Within the HIV-negative stratum, multidrug-resistant TB, including XDR-TB, is separated from active TB. XDR-TB is then separated from this broader multidrug-resistant category, leaving MDR-TB without extensive drug resistance. Drug-susceptible TB is obtained after excluding both MDR-TB without extensive drug resistance and XDR-TB from the corresponding HIV-negative active TB category. An analogous procedure is applied to HIV-associated TB to generate HIV-DS-TB, HIV-MDR-TB without extensive drug resistance, and HIV-XDR-TB.

Accordingly, DS-TB, MDR-TB without XDR, and XDR-TB constitute non-overlapping drug-resistance categories within each HIV stratum in the GBD 2023 modelling hierarchy. The same hierarchical definitions were retained throughout the present analysis to maintain consistency with the source estimates across the full 1990–2023 study period.

**S2.4. Interpretation of tuberculosis prevalence and latent tuberculosis infection**

GBD 2023 distinguishes latent tuberculosis infection from active tuberculosis disease. LTBI is defined as infection with Mycobacterium tuberculosis in the absence of symptoms or signs of active TB disease and is modelled separately using information including population-based tuberculin skin-test surveys and evidence on the risk of progression from infection to active disease.

In the GBD 2023 cause hierarchy represented in the data exports used for this study, the prevalence estimate for the parent tuberculosis cause (cause ID 297) includes latent tuberculosis infection (cause ID 954) in addition to the active drug-resistance subcategories. Therefore, the parent-level TB prevalence estimate should not be interpreted as the number of individuals with active TB disease. The high overall prevalence reported for this category primarily reflects the large population with latent M. tuberculosis infection.

For analyses specifically describing active drug-resistant disease, the corresponding GBD child causes—DS-TB (934), MDR-TB without XDR (946), and XDR-TB (947), together with the HIV-associated causes 948–950—were considered separately. This distinction was maintained when interpreting prevalence estimates in the Results and Discussion.
